# A Resolution of Identity Technique to Speed up TDDFT with Hybrid Functionals: Implementation and Application to the Magic Cluster Series Au_8*n*+4_(SC_6_H_5_)_4*n*+8_ (*n* = 3–6)

**DOI:** 10.1021/acs.jpca.3c05368

**Published:** 2023-10-31

**Authors:** Pierpaolo D’Antoni, Marco Medves, Daniele Toffoli, Alessandro Fortunelli, Mauro Stener, Lucas Visscher

**Affiliations:** † Dipartimento di Scienze Chimiche e Farmaceutiche, Università di Trieste, Via Giorgieri 1, Trieste 34127, Italy; ‡ CNR-ICCOM, Consiglio Nazionale delle Ricerche, via Giuseppe Moruzzi 1, Pisa 56124, Italy; § Department of Chemistry and Pharmaceutical Sciences, Vrije Universiteit Amsterdam, De Boelelaan 1083, Amsterdam 1081 HV, The Netherlands

## Abstract

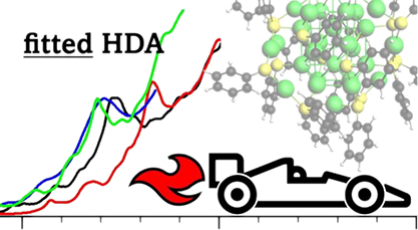

The Resolution of Identity (RI) technique has been employed to speed up the use of hybrid exchange-correlation (xc) functionals at the TDDFT level using the Hybrid Diagonal Approximation. The RI has been implemented within the polTDDFT algorithm (a complex damped polarization method) in the AMS/ADF suite of programs. A speedup factor of 30 has been obtained with respect to a previous numerical implementation, albeit with the same level of accuracy. Comparison of TDDFT simulations with the experimental photoabsorption spectra of the cluster series Au_8*n*+4_(SR)_4*n*+8_(*n* = 3–6; R = C_6_H_5_) showed the excellent accuracy and efficiency of the method. Results were compared with those obtained via the more simplified and computationally cheaper TDDFT+TB and sTDDFT methods. The present method represents an accurate as well as computationally affordable approach to predict photoabsorption spectra of complex species, realizing an optimal compromise between accuracy and computational efficiency, and is suitable for applications to large metal clusters with sizes up to several hundreds of atoms.

## 1 Introduction

Optical spectroscopies are among the most powerful investigation techniques in chemistry due to their direct applicability, easily accessible instrumentation, and ability to provide relevant information regarding the electronic structure of the studied system. However, it is often difficult to rationalize and assign the observed spectral features on the basis of purely experimental information. The availability of reliable models and accurate computational protocols therefore represents a formidable tool not only to rationalize the experiment but also to extract all the information, which is actually included in the experimental data but cannot be understood by a simple analysis of the spectrum.^1^ Moreover, if the computational protocol is able to reach quantitative accuracy, theory can be predictive, thus opening the way to the rational design, for example, of new materials with a required optical response. In this scenario, the best compromise between accuracy and computational economy is often the time-dependent density functional theory (TDDFT), which at the moment allows the calculation of systems containing up to 1000 of atoms. In quantum chemistry, the most popular implementation of TDDFT has been formulated by Casida.^2^ In the Casida formulation, an eigenvalue equation on the space of single-particle excitations, whose number is the product between the number of occupied and unoccupied orbitals, is solved. Besides the Casida approach, other TDDFT formulations and implementations are available.^3−11^ More recently, a complex polarizability algorithm for TDDFT^12−14^ (also known as polTDDFT) has proven particularly suitable for application to very large systems and will be employed also in the present work.^15^

In both DFT and TDDFT, accuracy represents a challenging issue, since it is intimately connected with the choice of the exchange-correlation (xc) functional in DFT to solve Kohn–Sham equations, and in the response kernel of TDDFT when solving the TDDFT equations.^16^ The search for more and more accurate xc functionals and kernels is a very active, widely spread, and highly demanded research activity.^17,18^ Hybrid xc functionals for DFT and hybrid kernels for TDDFT, i.e., functionals containing a variable fraction of the exact (Hartree–Fock, HF) nonlocal exchange, often represent the best choice in terms of accuracy, at least with respect to simpler local density approximation (LDA) and generalized gradient approximation (GGA). Among hybrid xc functionals, B3LYP^19,20^ is the most popular.

It is worth noting that in hybrid functionals, the HF nonlocal exchange can be problematic to handle when evaluating the matrix elements needed for the Casida equations. More precisely, matrix elements can be treated efficiently when Gaussian-type orbital (GTO) basis sets are employed but become problematic with other basis sets, such as slater-type orbitals (STO) or plane waves (PW). To solve these technical issues and get a still efficient TDDFT approach employing hybrid xc functionals, recently a new approximate scheme has been suggested for the TDDFT kernel, called Hybrid Diagonal Approximation (HDA).^21^ The HDA consists in employing the nonlocal exchange only for the diagonal terms in the response equations: this allows one to limit the computational cost of the TDDFT simulation while keeping almost the same accuracy as in the full TDDFT scheme using hybrid xc functionals.

The first HDA implementation, although much more efficient than the full-kernel one, was not fully optimized since the integrals needed to calculate the diagonal correction were calculated numerically. In the present work, we describe a much more efficient computational scheme to calculate such terms according to a procedure that avoids the numerical integration, by means of a density fitting auxiliary basis set and the Resolution of Identity (RI) technique. It is worth noting that auxiliary Gaussian–Hermite functions (named GEN-An GEN-An*)^22^ have proven to be generally useful as fitting functions; in fact, the same set is able to fit (1) the SCF Coulomb potential, (2) the SCF nonlocal exchange for hybrid functionals, and (3) the TDDFT-perturbed density.^23−26^ Use of STO functions for fitting is typically combined with the pair-fitting approximation^27,28^ since global fitting requires evaluation of three-center integrals for which for STOs, in contrast to GTOs, no analytical expressions are available.

In general, the RI technique has been introduced in modern quantum chemistry methods roughly 50 years ago in DFT^27^ and much later it started to be routinely employed in wave function theory (WFT) methods as well.^25,26,29−34^ In the RI approach, integrals are either approximated by a predetermined fit set or by an on-the-fly factorization procedure such as Cholesky decomposition.^35^ This reduces the computational scaling of computing multicenter integral contributions from a formal *n*^4^ to *n*^3^ or even lower depending on the approximations and distance screening techniques that are used. In the present work, since STO basis sets are employed, RI allows to avoid the numerical integration, which proved to be the bottleneck of the previous HDA implementation.^21^

The article is organized as follows. First we provide a short review of the HDA approximation in both the Casida and polTDDFT implementation. Then, we give the details of the RI method to calculate the HDA integrals as well as its implementation within the AMS-ADF program. In order to assess the performances of the method, we applied it to a series of four well-characterized gold clusters protected by aromatic thiolic ligands, with sizes between 268 and 460 atoms.^36^ Finally, a complete assessment of the RI method is given in terms of accuracy and computational efficiency, with respect to the numerical approach. A comparison with respect to photoabsorption experimental data as well as to the more approximate sTDDFT^7^ and TDDFT+TB^37^ approaches allows to assess the merits of the suggested approach.

## 2 Theoretical Method and Implementation

### 2.1 HDA

In order to briefly recall the HDA scheme,^21^ the most convenient way is to start with the random phase approximation (RPA) formulation of the linear response TDDFT approach:
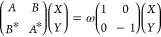
1where the submatrices *A* and *B* take the following general form for hybrid kernels:
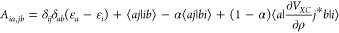
2
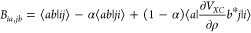
3where in equations 2 and 3, we employ *i* and *j* for occupied orbitals and *a* and *b* for the virtual ones. α represents the fraction of nonlocal HF exchange in the xc kernel, and the adiabatic local density approximation (ALDA) is assumed in the last terms of both equations. With STOs, it is very time-consuming to calculate the terms with the nonlocal exchange, namely, the third term in the rhs of equation 2 and the second term in the rhs of equation 3.

Now let us consider the diagonal elements of the RPA matrix, which correspond to the diagonal elements of matrix *A*:
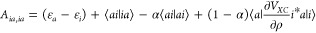
4

The third element in the right-hand side of equation 4 is the key ingredient to recover the too high occupied-virtual energy difference obtained from the KS equation when a hybrid xc functional, containing a fraction of HF exchange, is used. This simple observation suggests that a possible strategy is to employ the nonlocal HF exchange of the kernel only for the diagonal elements of the *A* matrix, while treating at the simpler ALDA level (no HF exchange) all the off-diagonal elements of the *A* matrix and the full *B* matrix. With this choice, which we named HDA,^21^ the matrices *A* and *B* take the following equations:
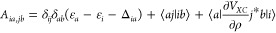
5
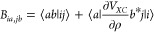
6where in eq 5, we have introduced a diagonal corrective term:
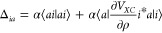
7

In eq 7, the second term is much smaller than the first term and can be safely neglected. With eq 5 and eq 6 for the *A* and *B* matrices, the difference *A–B* is still diagonal as it is customary in TDDFT with local kernels. Therefore, it becomes possible to solve the RPA-like equation with respect to *Y* and obtain again the conventional Casida equation for the Ω matrix, with the only difference occurring in the eigenvalue differences, which must be corrected by eq 7.

Importantly, following this approach, the number of required exchange integrals is only (Nocc × Nvirt) instead of (Nocc × Nvirt)^2^, i.e., it is greatly reduced. In the previous implementations of eq 7, the first term was calculated numerically while the second term is neglected.

### 2.2 Extension of HDA to the Complex Polarizability TDDFT Formulation

Despite the efficiency of numerical diagonalization techniques such as the Davidson one, it becomes hard to calculate valence photoabsorption spectra over a wide excitation energy range when large systems are considered. The Davidson iterative algorithm, generally employed in all the TDDFT codes that use the Casida method, is efficient on large Ω matrices but is limited to extracting a relatively small number of lowest eigenvalues and eigenvectors. In order to overcome this problem, we have recently proposed the complex polarizability TDDFT (polTDDFT) algorithm.^12^ This approach avoids the diagonalization bottleneck via a direct solution of the response equations and thereby allows for the treatment of very large systems. The reader is referred to the original work for a detailed description of the algorithm,^12^ together with its implementation in the ADF program.^13^

In polTDDFT, the photoabsorption spectrum σ(ω) is discretized and computed in a limited number of points from the imaginary part of the dynamical polarizability α(ω):
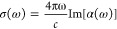
8

The polarizability is calculated for complex frequencies, i.e., ω = ω_r_ + *i*ω_i_, where the real part ω_r_ is the scanned photon frequency (energy) and ω_i_ is the imaginary part, which corresponds to a broadening of the discrete lines and can be interpreted as a pragmatic inclusion of the excited-state finite lifetime. The complex dynamical polarizability is calculated by solving the following nonhomogeneous linear system:
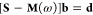
9

In eq 9, **S** is the overlap matrix between fitting functions, **b** is the unknown vector with the expansion coefficients *b*_μ_(ω) of the induced density ρ_*z*_^(1)^, **d** is the frequency-dependent vector corresponding to the known nonhomogeneous term, and finally the elements of the frequency-dependent matrix **M** are
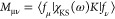
10

In eq 10, χ_KS_ refers to the Kohn–Sham frequency-dependent dielectric susceptibility and *K* to the kernel. The original characteristic of the polTDDFT method is the introduction of a simple approximation, which enables the construction of **M**(ω) as a linear combination of *frequency-independent* matrices **G**^*k*^ with frequency-dependent coefficients *s*_*k*_(ω), with the following equation:
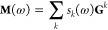
11With this idea, a set of matrices {**G**^*k*^} is calculated and stored only once at the beginning and then the matrix **M**(ω) is calculated very rapidly at each photon energy ω, as a linear combination of the {**G**^*k*^} matrices with the following coefficients:
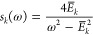
12where in equation 12, *E̅*_*k*_ refers to the center of the interval, which discretizes the excitation energy variable and in the original formulation corresponds to the difference between virtual and occupied orbital energies: ε_a_–ε_i_.

In order to apply within the polTDDFT algorithm the HDA approach already discussed for the RPA procedure, it is sufficient to correct the orbital energy differences with the same correction term (7) as already done for equation 5: ε_a_–ε_i_–Δ_ia_.

### 2.3 Resolution of Identity implementation (Fitted HDA)

As mentioned in the previous section, in the previous HDA implementation, the first term of the diagonal correction (7) was calculated numerically, which is accurate but still time-consuming although much less demanding than the complete nonlocal kernel. Finding a cheaper way to get equation 7 that avoids the time-consuming numerical integration is therefore desirable. We can do so by expressing the two-electron integral of eq 7 as a sum of analytical integrals through the RI technique in which the density of each molecular orbital is expressed as a linear combination of auxiliary density fitting functions. We start by rewriting the corrective diagonal integral as follows, taking into account that the molecular orbitals are real so that complex conjugation of the bra functions can be omitted:
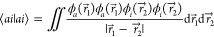
13

The numerator of the integrand is just the product of the electron densities of orbitals ϕ_a_ and ϕ_i_:
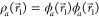
14
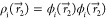
15

Both densities can be approximated as a sum of density fit functions as
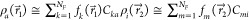
16

Using the above equations to rewrite eq 13, we get:
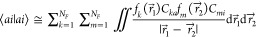
17

For convenience, we define a matrix *F*, with dimension of the auxiliary density fitting basis set, whose elements are
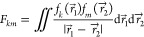
18

The key step feature of this RI approach is that an analytical expression can be found for the integral (18) even for STO functions located at different centers. After computing *F,* the corrective diagonal integrals can be easily calculated via the matrix transform:
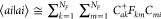
19once the *C* coefficients have been determined. These follow from the projection of the orbital density ρ_*a*_(*r⃗*_1_) on the set of fit functions *f*_*p*_:
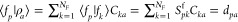
20where *S*^f^ is the overlap matrix between fitting functions pairs. In this work, we follow the standard choice of using the overlap metric in the determination of fit coefficients; an alternative is to use robust fitting in which the Coulomb metric is used to define the scalar product ⟨*f*_*p*_ | ρ_*a*_⟩. An extensive discussion of different fit procedures for STOs and their accuracy can be found in ref (28). Solving the linear set of eq 20 yields the fit coefficients for the virtual orbital densities, and a similar procedure is followed for the occupied orbitals φ_i_ as well. We have
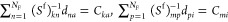
21so that we can rewrite eq 19:
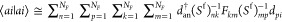
22

The RI approach makes it possible to build and store a new matrix *Q*, to also include the second correction term of equation 7, which was neglected in the previous numerical implementation. In Section 4.1, we analyze the effect of the second term, which actually gives negligible contribution, justifying the previous choice, in the numerical implementation of HDA, to neglect this term. In fact, now it can be easily included since the matrix of the ALDA kernel over the auxiliary density fitting basis is already available since is needed by the polTDDFT (see matrix ***Z*** in ref (12)):
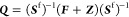
23

Finally, the correction for the diagonal term is obtained as
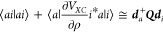
24

### 2.4 Implementation of Efficient HDA

In the AMS implementation, many of the required matrices and integrals needed for this purpose are already available. We first consider the calculation of the *Q* matrix. The auxiliary **L** matrix is defined as
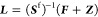
25and computed, taking advantage of Scalable Linear Algebra PACKage (ScaLAPACK), by solving the following linear system
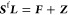
26Thus, taking advantage of the already existing **L** and **S** matrices, we can express **Q** as
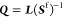
27

To compute it, we can multiply the above equation by ***S***^f^ on the right side and then take the transpose of both sides. Then, considering that both **Q** and **S** are real symmetric matrices, we thus obtain the following linear system whose formal solution is the **Q** matrix; to solve it, we just use the same routines used before in the code to obtain the **L** matrix.
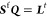
28

This implementation could be realized directly inside a pre-existing (CalcMatrixL.f90) subroutine. We needed to transpose and store the **L** matrix. Regarding the calculation of *d*_*a*_ and *d*_*i*_ vectors, this is done in a new, appositely created, routine. This new routine has been built starting from an already existent one, which calculates the integrals between a fit function and a product of a pair of occupied–virtual molecular orbitals. This was modified, changing the occupied–virtual products with occupied–occupied and virtual–virtual products.

Regarding the representation of the matrices, the matrices of the polTDDFT method (eqs 9-12) are in the density fitting basis as well as matrix F (eq 18) as well as the matrices in eqs 25-28. The RPA matrices are well-known and are in the basis of the MO products occupied*virtual (the so-called 1h1p space in the Configuration Interaction method).

As an outcome of this section, we conclude that we have been able to exploit in AMS the efficient RI technique and convert the numerical calculation of diagonal corrective term (eq 7) to a sum of analytical integrals.

## 3 Computational Details

All the DFT KS calculations reported in this work were performed employing a TZP basis of STO functions (included in the ADF database) and the B3LYP hybrid xc functional.^19,20^ The auxiliary density fitting basis STO functions are taken from the POLTDDFT basis set database included in the AMS program.^38^ The TDDFT calculations were performed with the polTDDFT algorithm.^12,13^ The HDA-B3LYP density functional approxiamtion^21^ is the only available scheme for hybrid functional within polTDDFT. The HDA has been implemented by exploiting the parallelization at the general Message Passing Interface (MPI) level. The imaginary part of the photon energy (see ω_*i*_ after eq 8) has been set to 0.075 eV during the calculation of the polTDDFT spectra. All of the calculations were performed with a local version of the ADF code, which will be distributed in a forthcoming ADF release. The calculations for the gold cluster in Section 4 were performed employing the zero-order regular approximation (ZORA)^39^ in order to include relativistic effects at the scalar level. In order to have a comparison with an alternative, much cheaper computational scheme, we have also performed sTDDFT^7^ and TDDFT+TB calculations^37^ on the same metal clusters. All the calculations have been performed on an HPE ProLiant DL580 Gen10 server (with four processors each with an 18-core Intel Xeon Gold 6140 CPU at 2.30 GHz, in total 72 cores and 728 GB of RAM), on which the present calculations were run using 24 cores. The coordinates of the clusters were taken from the experiment, but ligands have been simplified to benzene thiol instead of *tert*-butylbenzene thiol. The coordinates of the four clusters are reported in Tables S1 and S4 of the Supporting Information.

## 4 Results and Discussion

**Figure 1:**
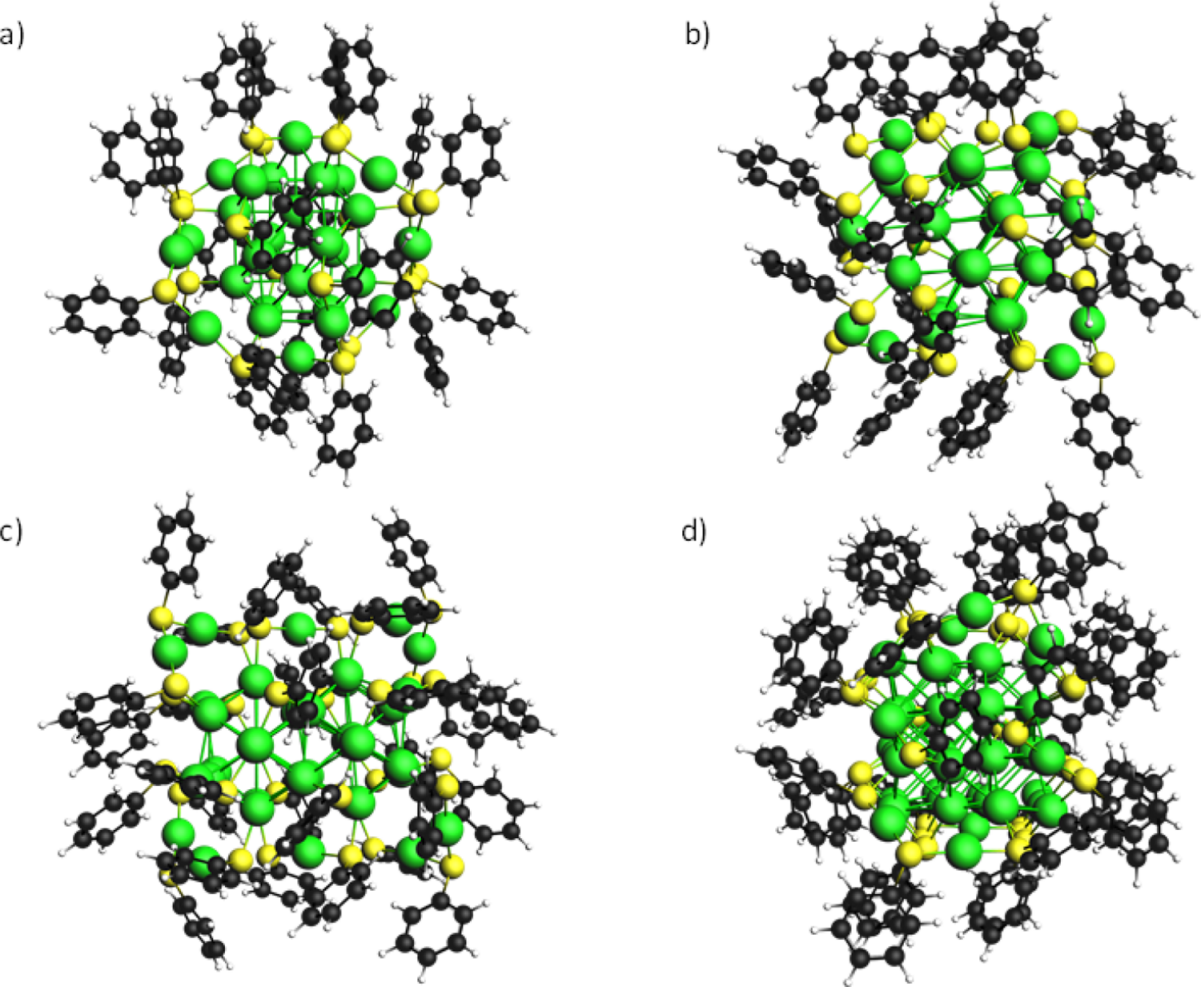
Cluster series experimental geometries with simplified ligands: (a) Au_28_(SC_6_H_5_)_20_, (b) Au_36_(SC_6_H_5_)_24_, (c) Au_44_(SC_6_H_5_)_28_, and (d) Au_52_(SC_6_H_5_)_32_.

In order to test the performance and assess the accuracy of the RI-fitted HDA algorithm, the photoabsorption spectra of a gold cluster series^36^ have been simulated and compared with the available experimental data. Moreover, the results obtained with the new RI algorithm were also compared with those simulated using the already available numerical-integration HDA,^21^ the sTDDFT,^7^ and the more simplified and cheaper TDDFT+TB^37^ methods. This cluster series is of particular interest because it represents a typical “magic series” with formula Au_8*n*+4_(SR)_4*n*+8_(*n* = 3–6; R = C_6_H_5_). Figure 1 shows the geometries of the clusters, which were taken from the X-ray diffraction experiment^36^ but have been simplified using benzene instead of *tert*-butylbenzene residues in the thiols. This simplification has no impact on the photoabsorption spectra and allows to lighten the computational cost of the simulation, as proved by previous work.^40^ Moreover, the same simplification of the ligands has been already employed previously to simulate the same magic series.^41^

From Figure 1, it is possible to appreciate how the organic layer tends to reduce its conformational energy by maximizing the stabilizing interactions; for example, phenyl groups are disposed face to face to allow the π–π stacking interaction between delocalized aromatic electrons.

### 4.1 Au_28_(SC_6_H_5_)_20_

**Figure 2:**
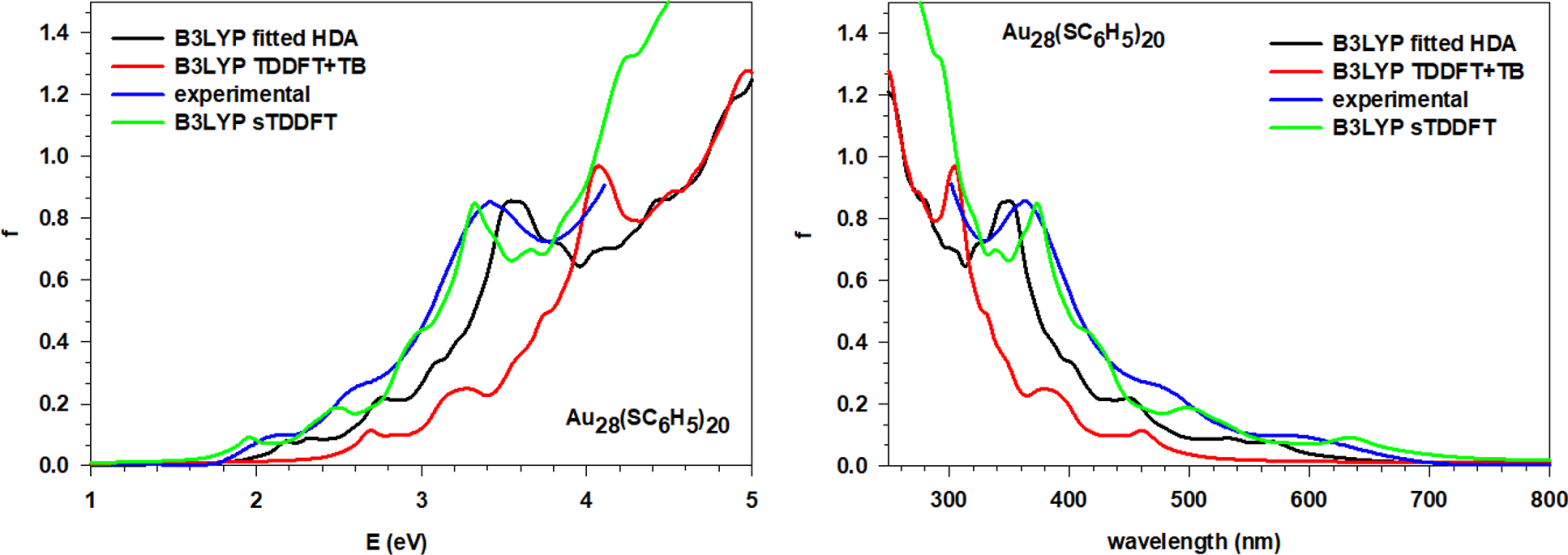
Comparison of the experiment with the simulated spectra using fitted HDA, sTDDFT, and TDDFT+TB for the Au_28_(SC_6_H_5_)_20_ cluster, both energy (eV), above, and wavelength (nm), below, plots are reported.

In the following, we will focus on the smaller cluster of this series, Au_28_(SC_6_H_5_)_20_. The comparison between the experimental and the simulated spectra, obtained using fitted HDA, TDDFT+TB, and sTDDFT schemes, for this cluster is reported in Figure 2. It is clear that fitted HDA is able to reproduce very nicely the experimental data on the whole energy range available (up to 4.25 eV). In particular, the strong peak at 3.4 eV of the experiment is well reproduced by the fitted HDA scheme with a limited overestimation in the excitation energy of only 0.14 eV. The performance is similar for the other spectral features, which are less intense and occur at lower energy values: their excitation energies and relative intensities with respect to the experiment are also nicely reproduced. On the other hand, the TDDFT+TB method reproduces correctly the spectral shape but yields strong overestimation of the excitation energy, by almost 1 eV. It is worth noting, however, that above 4 eV the TDDFT+TB and fitted HDA profile are in excellent match. This suggests that TDDFT+TB is able to reproduce correctly the high energy part of the spectrum, a spectral region in which the ligands play a major role. At lower energies, where the metal excitations dominate, the tight-binding approximation is not as good and the more accurate fitted HDA scheme performs much better with a predictive quantitative accuracy. On the other hand, the sTDDFT scheme is in fairly nice agreement with the experiment, with an accuracy superior to that of the fitted HDA with regard to the shape of the profile reported in Figure 2. The energy of the maximum of the experimental absorption falls at 3.43 eV, while it is calculated at 3.32 and 3.56 eV with sTDDFT and fitted HDA, respectively, with a discrepancy around 0.1 eV for both methods.

**Figure 3:**
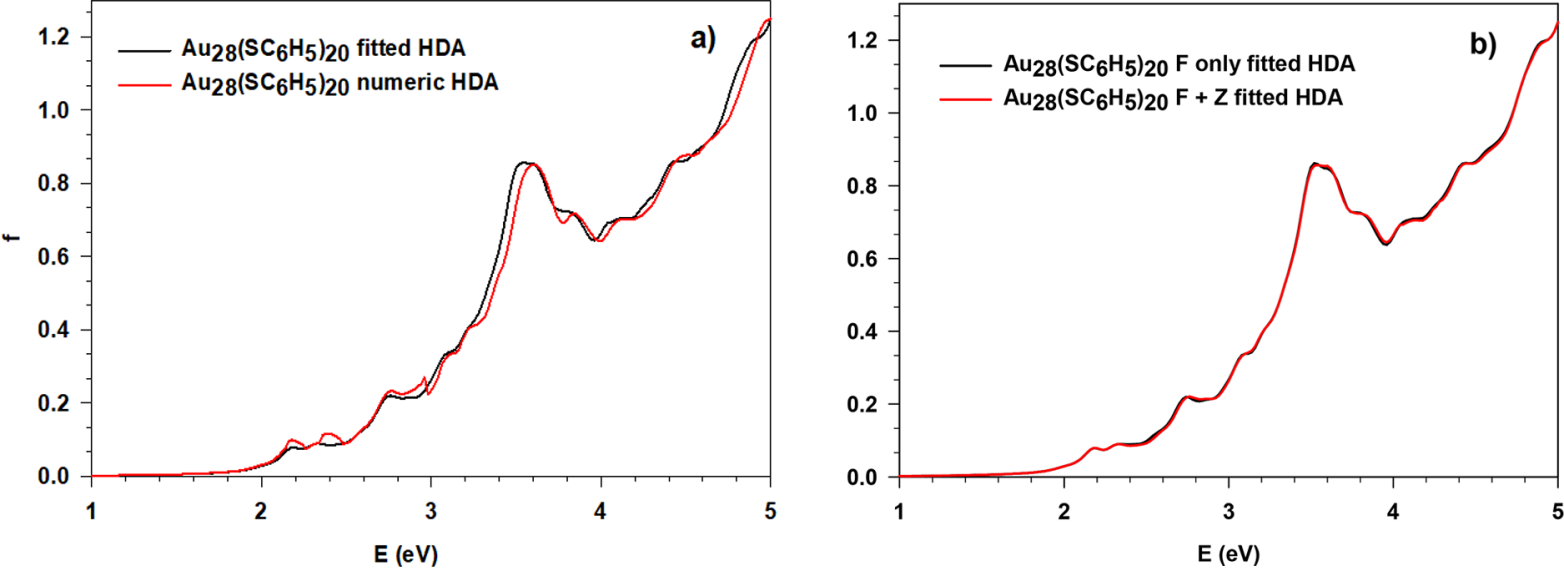
Comparison of the photoabsorption spectra for the Au_28_(SC_6_H_5_)_20_ cluster with polTDDFT. (a) Numeric HDA and new fitted HDA. (b) New fitted HDA with only *F* and complete *F*+*Z* kernel (see eq 23).

The accuracy of numeric HDA has been already tested in a previous work^21^ so, to demonstrate the benefit of using the new fitted approach in HDA, in terms of both accuracy and computational economy, the numeric HDA calculation for this smallest cluster has been performed as well. The spectra obtained with the two different approaches to HDA are reported in Figure 3a, and it is clear how both methods lead to the same simulated spectrum with negligible differences. We employed this cluster also to verify if the approximation previously introduced in the numerical HDA, namely, to neglect the fraction of the XC ALDA kernel in the diagonal correction, is justified. With the fitted HDA, this task is trivial; it consists of using only the *F* matrix instead of *F*+*Z* in eq 23. The comparison is reported in Figure 3b, and the two profiles are almost superimposed so the role of the *Z* matrix is marginal and the previous approximation is justified.

The accuracy of fitted HDA in comparison with standard numeric HDA, Figure 3, and with experiment, Figure 2, takes on even greater significance when considering the times needed for the simulations. For this smaller cluster, Au_28_(SC_6_H_5_)_20_*,* of “only” 268 atoms, it takes 83 h using numeric HDA and 3 h using the new fitted approach. In other words, fitted HDA allows to obtain the same photoabsorption spectrum as numeric HDA but 28 times faster. For comparison, the sTDDFT calculation on the same cluster took 55 h while TDDFT+TB only 11 min. From this analysis, it is evident that TDDFT+TB is 1 order of magnitude faster than fitted HDA but much less accurate; on the other hand, sTDDFT displays a comparable accuracy as fitted HDA but is less efficient by one order of magnitude. Similar relative behaviors between the method timings have been obtained for the larger clusters as well; however, since the timings are somehow dependent on the machine load, we do not report their values. From this simple analysis, it seems that a still more efficient scheme could be obtained integrating sTDDFT with polTDDFT: for example, using the very efficient sTDDFT method to build the matrix elements but employing the polTDDFT algorithm to solve the TDDFT equations.

This is a remarkable speedup, which enabled us to simulate and study the remaining, larger clusters of the series using the polTDDFT-HDA scheme in a reasonable amount of CPU time (less than 1 week). The simulations for each cluster of the series were then characterized by analyzing the most prominent spectral features in terms of one-electron-excited configurations, i.e., in pairs of occupied–virtual molecular orbitals. Moreover, the nature of the molecular orbitals involved in the electronic transitions was also analyzed via Mulliken population analysis and direct inspection of the plotted orbitals.

**Figure 4:**
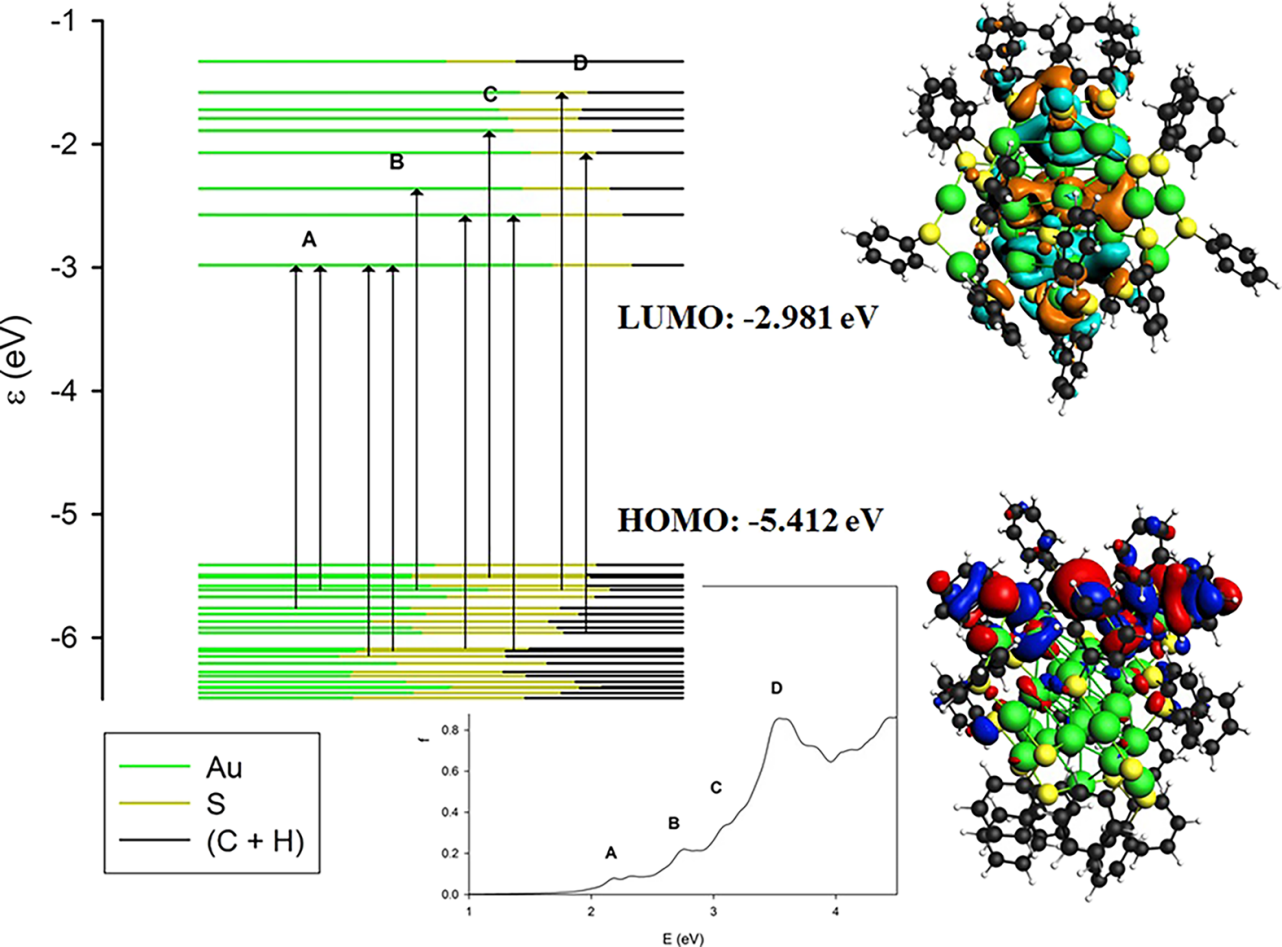
On the left, the molecular orbital energy plot for the Au_28_(SC_6_H_5_)_20_ cluster with arrows indicating the major transitions involved in each spectral feature (A, B, C, and D), on the right, HOMO and LUMO energies, and orbital plots. The contribution of different fragments (the metal core of Au atoms, the ligand’s polar heads S, and their spacers C+H) to each molecular orbital considered is depicted through the ratio of the associated colors.

Let us continue to consider the Au_28_(SC_6_H_5_)_20_ cluster. From the analysis of the molecular orbital energy levels, we find that the HOMO lies at −5.412 eV while the LUMO lies at −2.981 eV leading to an energy gap of 2.431 eV. This band gap is quite large, confirming that this system has a closed-shell electronic structure and is particularly stable, as expected for a member of a magic series. The major spectral features are the four peaks labeled A, B, C, and D in the side panel of Figure 4 and whose main contributions in terms of one-electron excited configurations are reported in Table 1. It is worth noting that feature A displays a strong contribution for the most relevant one-electron excited configuration (62% HOMO–6 → LUMO) and can be qualitatively described with only one excited configuration. In contrast, the following transitions at higher energies (A, B, and C) cannot be described with only one configuration, since the maximum contribution is around 21% for B and C and even 11% for D, indicating that they must be described as a mixing of several configurations. From the analysis of the orbital contributions, it is worth noting that the occupied orbitals close to the HOMO have a very mixed character with both the metal atoms and ligands contributing, while virtual orbitals show a more pronounced metal contribution (as apparent from the different proportions of each color in the molecular orbitals energy plot of Figure 4). A visual representation of this behavior is in the HOMO and LUMO plots of Figure 4, while the plots of all the most relevant molecular orbitals are provided in the Supporting Information in Figures S3 and S4.

**Table 1:** Principal Contributions in Terms of One-Electron Excited Configurations to the Main Spectral Features of Au_28_(SC_6_H_5_)_20_ Calculated at PolTDDFT Level with the B3LYP XC Functional and TZP Basis Set and Using Fitted HDA

**excitation**	**excitation *E* (eV)**	***f***	**assignment**
A	2.34	0.0898	62.53% HOMO–6 (21% S 3p, 8% Au 5d, 5% C 2p)→ LUMO (25% Au 6p, 22% Au 6s, 2% S 3p); 18.25% HOMO–4 (20% Au 6s, 13% S 3p, 6% Au 5d) → LUMO
			
B	2.76	0.2206	21.5% HOMO–14 (25% S 3p, 4% C 2p, 1% Au 6s) → LUMO; 18.21% HOMO–13 (14% S 3p, 11% C 2p, 5% Au 5d) → LUMO; 10.97% HOMO–4 → LUMO+2 (17% Au 6p, 16% Au 6s, 7% S 3p)
			
C	3.10	0.3383	21.23% HOMO–11 (22% S 3p, 4% Au 6s, 4% C 2p) → LUMO+1 (21% Au 6p, 19% Au 6s); 15.85% HOMO–2 (28% S 3p, 16% Au 5d, 3% Au 6s) → LUMO+4 (21% Au 6p, 14% Au 6s, 3% S 3p); 10.97% HOMO–13 → LUMO+1
			
D	3.54	0.8568	10.94% HOMO–4 → LUMO+7 (25% Au 6p, 8% Au 6s, 1% C 2p); 10.59% HOMO–10 (15% S 3p, 8% Au 6s, 4% Au 5d) → LUMO+3 (24% Au 6p, 18% Au 6s, 2% S 3p)
			

It is worth noting that the intensities of the spectral features increase with energy. This is a quite general behavior for these systems, and it is due to the increasing density of excited states with energy. In the specific case, it can be attributed to the manifold of empty orbitals that accept the excited electron. In fact, from Figure 4, it is well apparent that the set of occupied orbitals from which the transitions originate for the different spectral features A, B, C, and D are roughly the same while the virtual “arrival” orbitals cover a wider energy range. From this analysis, it is possible to attribute all these features to transitions from Au–S bonding orbitals to orbitals belonging mainly to the cluster metal core, so that these transitions have a partial character of ligand-to-metal charge transfer. In particular, the D feature is rather intense. This can also be related to the aromatic nature of the ligands; in fact, we have previously shown^40^ that aromatic ligands can amplify the transition intensity by an electronic coupling mechanism between the metal cluster and the ligand through a conjugation effect.

The induced density and independent component mapping of oscillator strength (ICM-OS) plots were also inspected in order to identify possible plasmonic behaviors and the connection between absorption and single-particle excitations.^42^ However, no such collective behavior has been detected. The plots are available in Supporting Information in Figure S1. The absence of collective behavior follows from the absence of contributions (spots), which lie far from the white straight line corresponding to occupied–virtual orbital pairs whose energy difference equals the photon energy in the ICM-OS plots. The corresponding induced densities are reported in Figure S2, and also in this case one can observe that there is no indication of a plasmonic behavior. Indeed, plasmons usually give induced densities with an evident dipolar shape whereas the plots reported in Figure S2 display very irregular shapes, which cannot be ascribed to dipolar shapes at all.

### 4.2 Au_36_(SC_6_H_5_)_24_, Au_44_(SC_6_H_5_)_28_, and Au_52_(SC_6_H_5_)_32_

**Figure 5:**
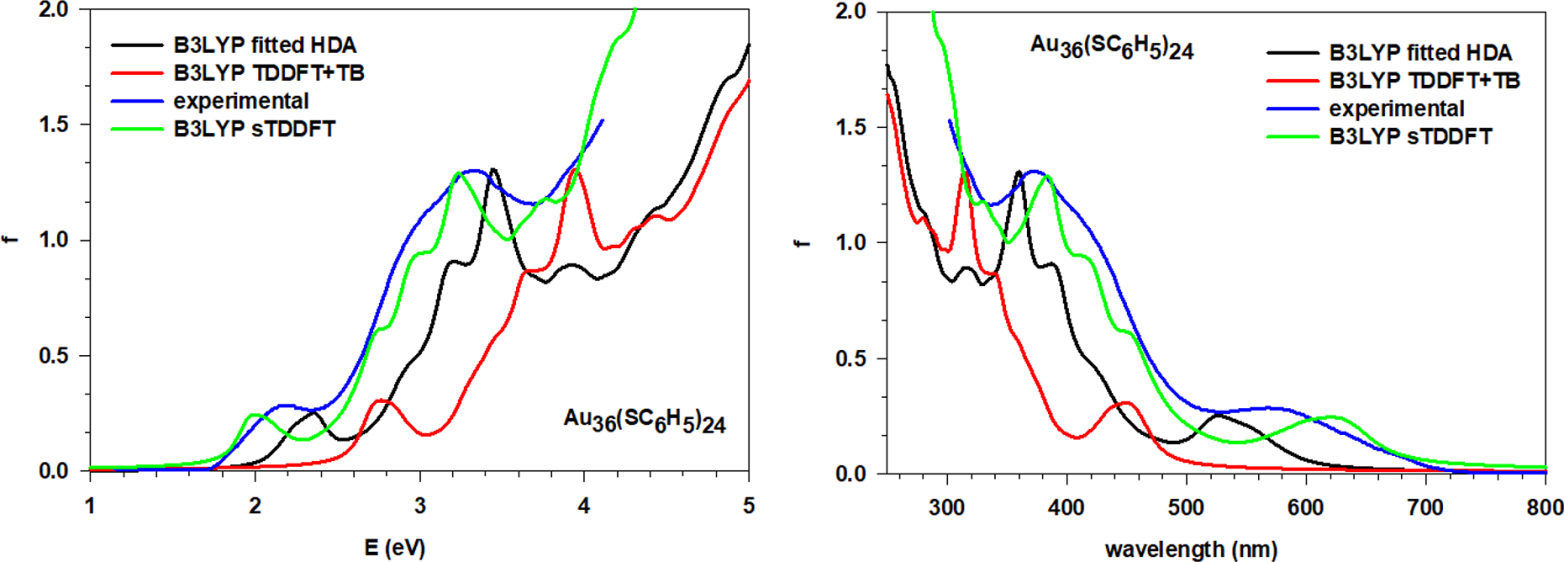
Comparison of the experiment with the simulated spectra using fitted HDA, sTDDFT, and TDDFT+TB for the Au_36_(SC_6_H_5_)_24_ cluster; both energy (eV), left, and wavelength (nm), right, plots are reported.

**Figure 6:**
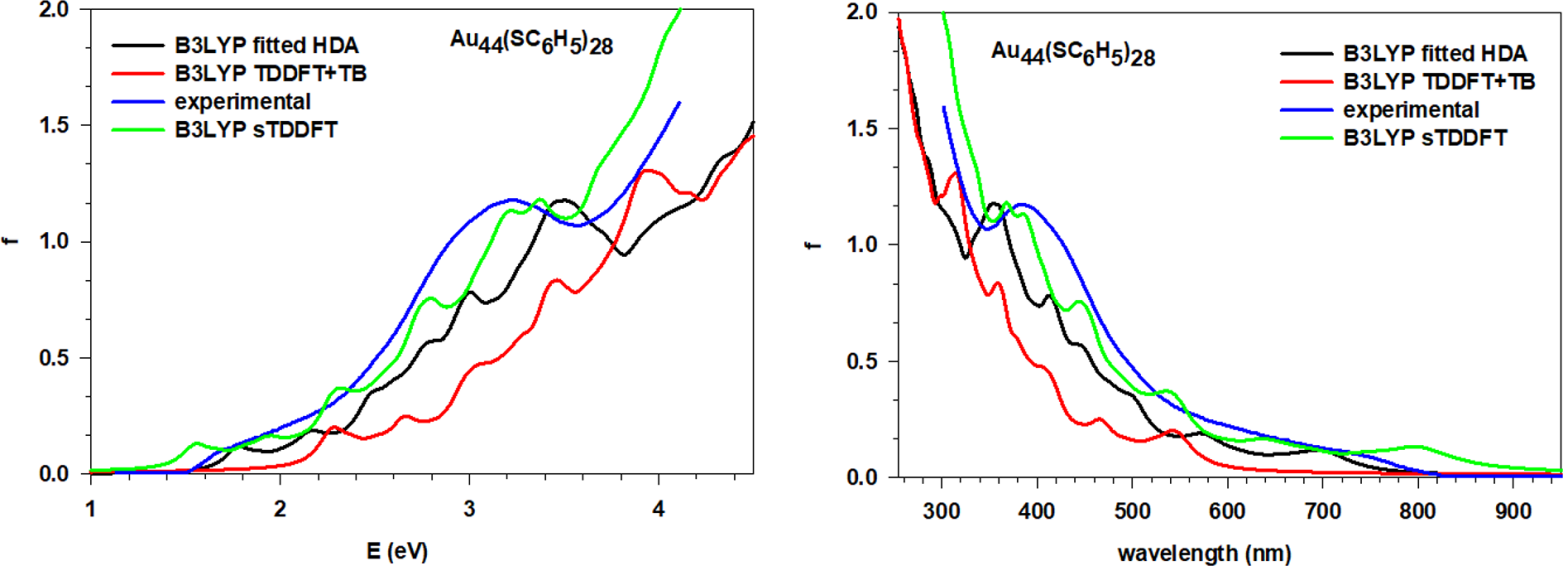
Comparison of the experiment with the simulated spectra using fitted HDA, sTDDFT, and TDDFT+TB for the Au_44_(SC_6_H_5_)_28_ cluster; both energy (eV), left, and wavelength (nm), right, plots are reported.

**Figure 7:**
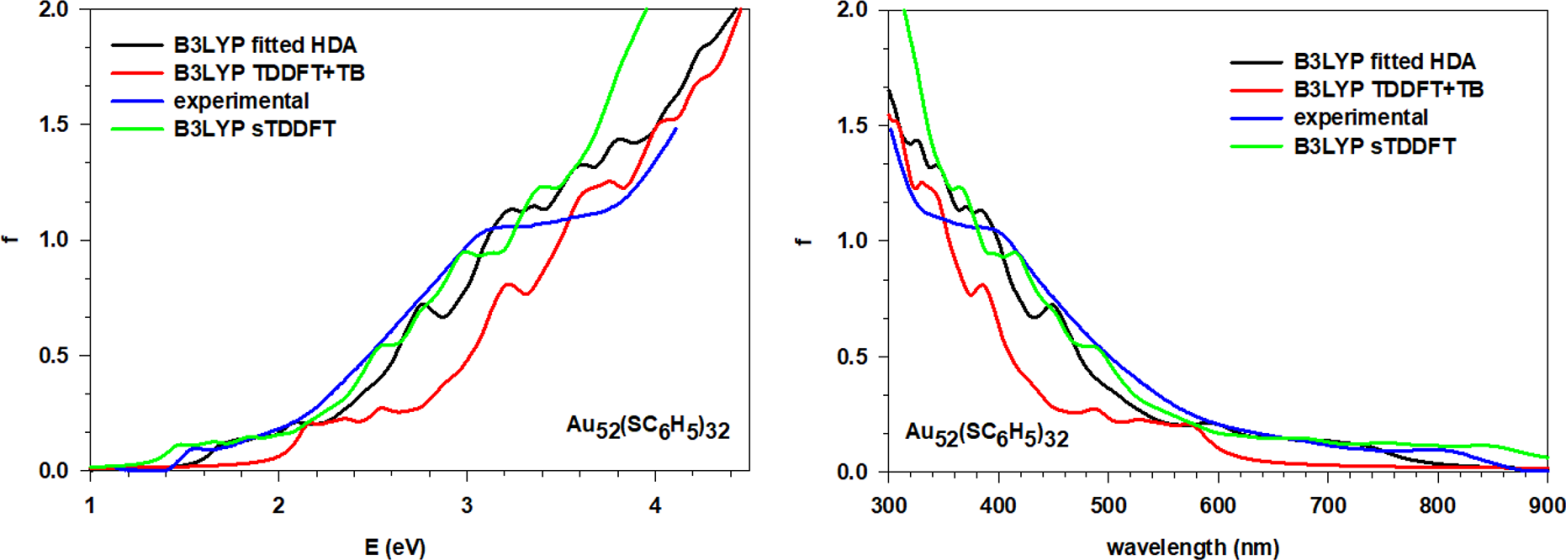
Comparison of the experiment with the simulated spectra using fitted HDA, sTDDFT, and TDDFT+TB for the Au_52_(SC_6_H_5_)_32_ cluster; both energy (eV), left, and wavelength (nm), right, plots are reported.

**Figure 8:**
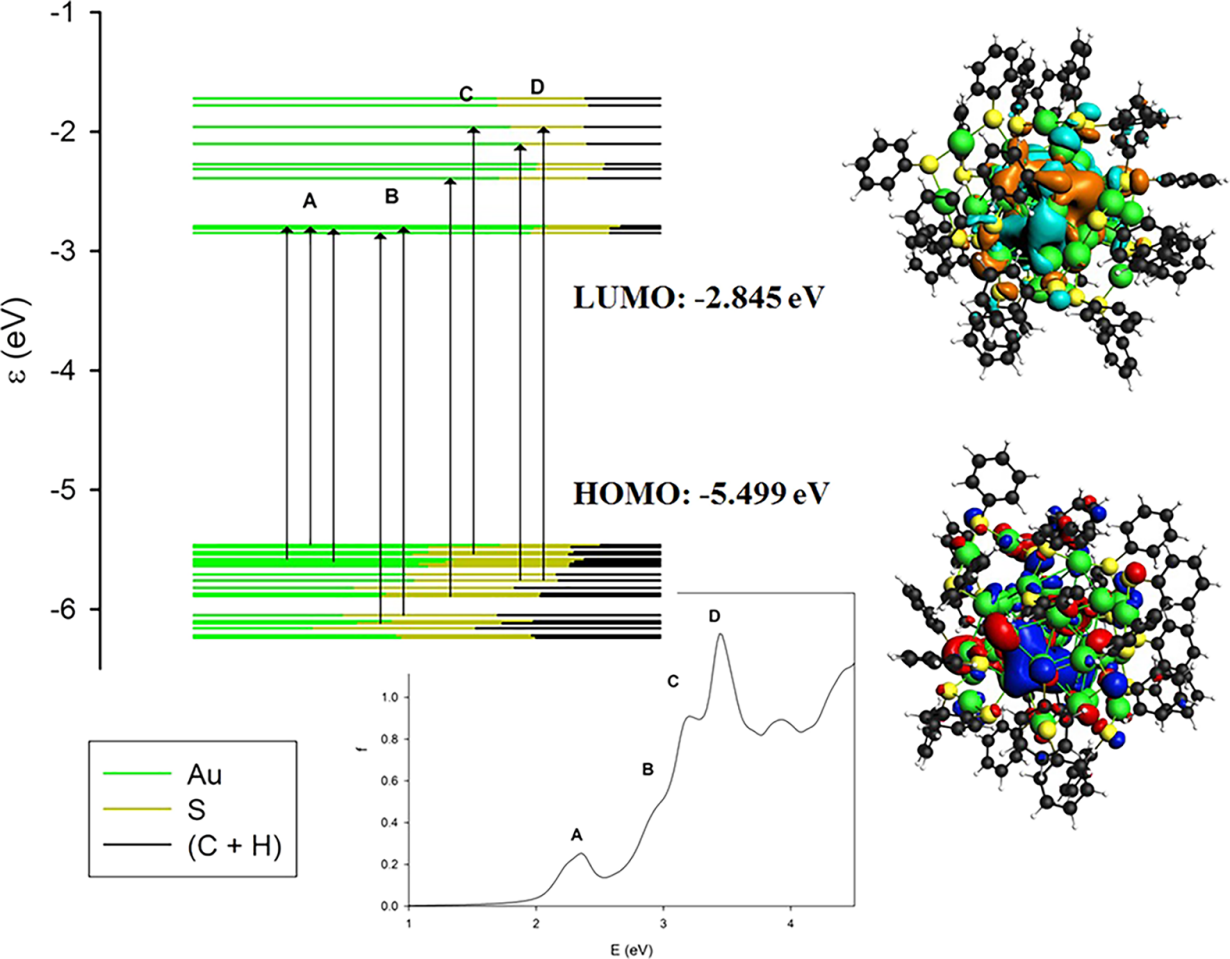
On the left, the molecular orbital energy plot for the Au_36_(SC_6_H_5_)_24_ cluster with arrows indicating the major transitions involved in each spectral feature (A, B, C, and D), on the right, HOMO and LUMO energies and orbital plots. The contribution of different fragments (the metal core of Au atoms, the ligand’s polar heads S, and their spacers C+H) to each molecular orbital considered is depicted through the ratio of the associated colors.

**Figure 9:**
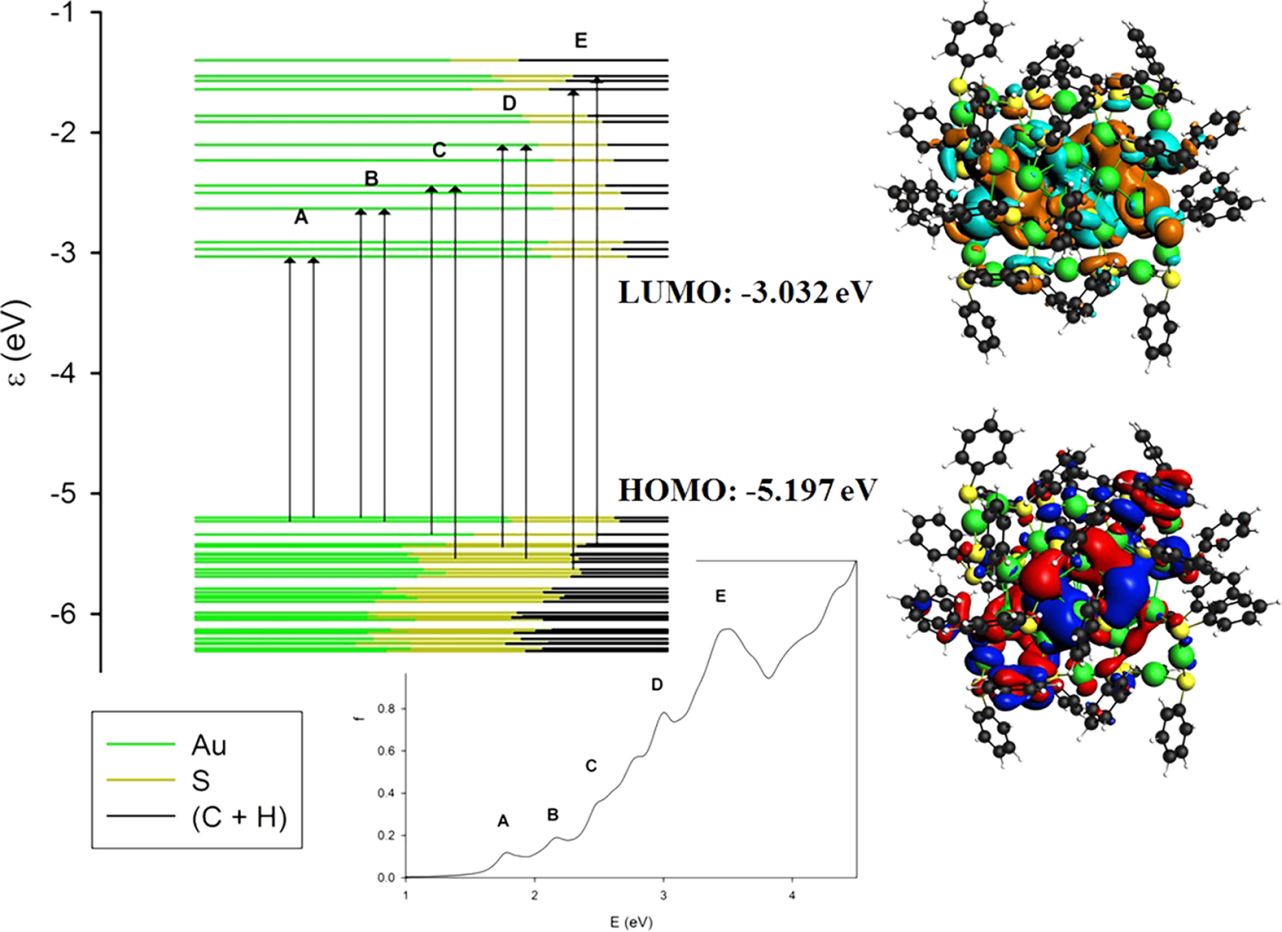
On the left, the molecular orbital energy plot for the Au_44_(SC_6_H_5_)_28_ cluster with arrows indicating the major transitions involved in each spectral feature (A, B, C, D, and E), on the right, HOMO and LUMO energies and orbital plots. The contribution of different fragments (the metal core of Au atoms, the ligand’s polar heads S, and their spacers C+H) to each molecular orbital considered is depicted through the ratio of the associated colors.

**Figure 10:**
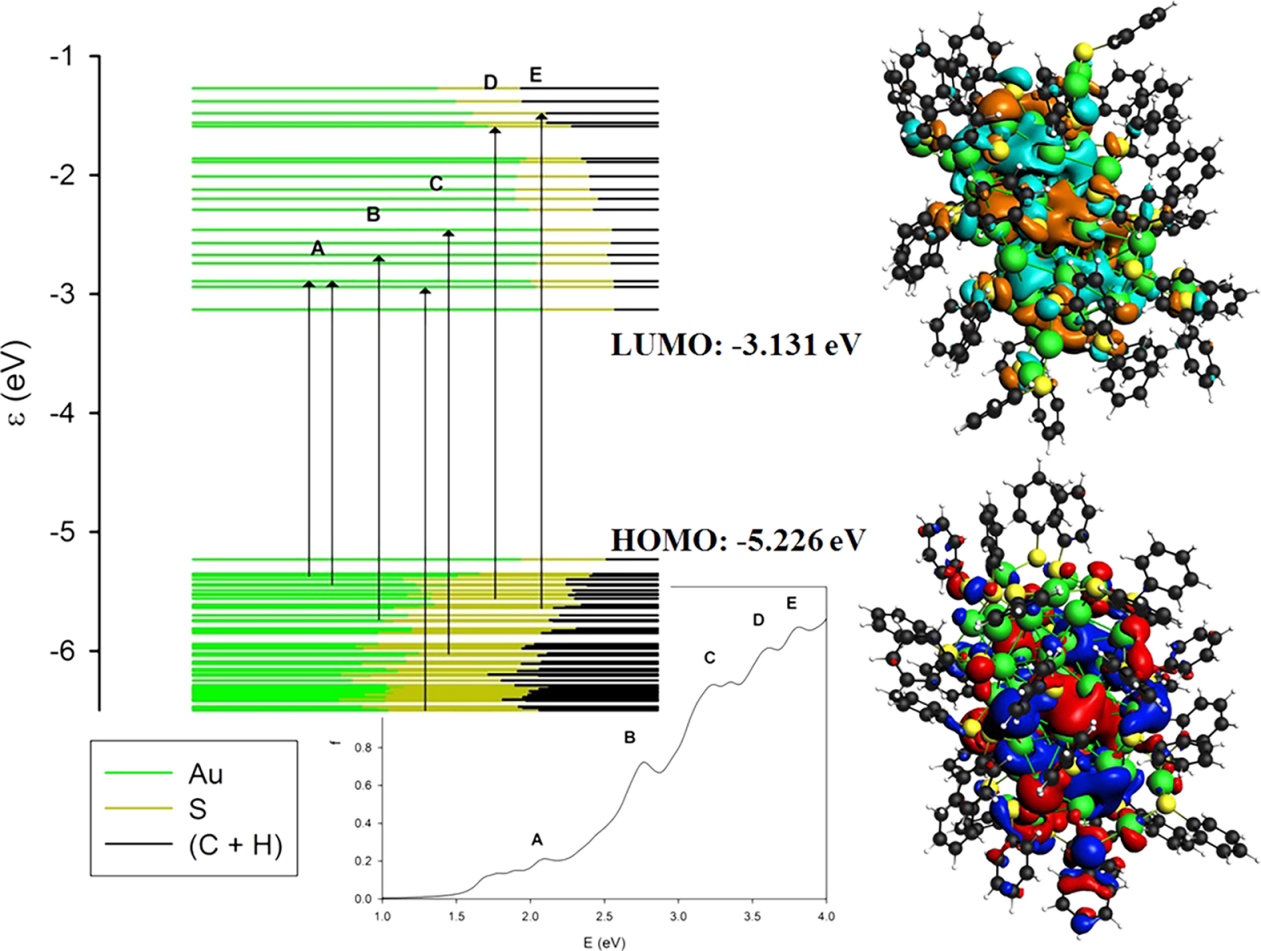
On the left, the molecular orbital energy plot for the Au_52_(SC_6_H_5_)_32_ cluster with arrows indicating the major transitions involved in each spectral feature (A, B, C, D, and E), on the right, HOMO and LUMO energies and orbital plots. The contribution of different fragments (the metal core of Au atoms, the ligand’s polar heads S, and their spacers C+H) to each molecular orbital considered is depicted through the ratio of the associated colors.

The same analysis has been performed for all of the remaining clusters of the series with analogous results. For each member of the series, we have considered the comparison between the photoabsorption spectra simulated using fitted HDA, sTDDFT, and TDDFT+TB with respect to the experimental data (Figures 5, 6, and 7), the molecular orbital energy plots (Figures 8, 9, and 10), and the analysis in terms of one-electron excited configurations of the main spectral features (Tables 2, 3, and 4). Considering each cluster separately, it is clear how the simulations using fitted HDA are in better agreement with the experiment when compared to the simulations using TDDFT+TB, while sTDDFT displays an accuracy comparable to fitted HDA. The location of the absorption peaks retraces almost perfectly that of the experiment with minor discrepancies of few tenths of eV. The general profile is also well reproduced with the correct trend for the absorption intensities and its peculiarities, like the two more pronounced peaks of Au_36_(SC_6_H_5_)_24_ (Figure 5) near 2.25 and 3.5 eV. The simulations obtained with the TDDFT+TB scheme all show a positive energy shift of ∼0.5 eV. Although we observe a smooth trend along the series of the spectra, there are some interesting discontinuities such as, for example, the just noted feature of Au_36_(SC_6_H_5_)_24_ at 2.25 eV. This effect resembles the previously observed “rebirth of the plasmon” in Au_30_(SR)_18_,^40^ which was ascribed to a strong electronic conjugation between the metal core and the aromatic ligands. Therefore, we are led to conclude that in present series such conjugation is maximized in the Au_36_(SC_6_H_5_)_24_ cluster, Au_28_(SC_6_H_5_)_20_ being too small while Au_44_(SC_6_H_5_)_28_ and Au_52_(SC_6_H_5_)_32_ probably are too congested (due to their larger radius), and therefore, their structures do not allow proper conjugation between the ligands and the metal core. Another peculiarity of Au_36_(SC_6_H_5_)_24_, which can be correlated with the strong feature at 2.25 eV, is the presence of many virtual orbitals almost degenerate with the LUMO (see Figure 8). By comparison with Figures 3, 9, and 10, it is well apparent that while in the other clusters the virtual orbitals are distributed rather uniformly above the LUMO, whereas for Au_36_(SC_6_H_5_)_24_ such distribution is much less homogeneous.

**Table 2:** Principal Contributions in Terms of One-Electron Excited Configurations to the Main Spectral Features of Au_36_(SC_6_H_5_)_24_ Calculated at the PolTDDFT Level with the B3LYP XC Functional, TZP Basis Set, and Using Fitted HDA

**excitation**	**excitation *E* (eV)**	***f***	**assignment**
A	2.36	0.2533	17.51% HOMO–4 (16% S 3p, 8% Au 6s, 6% Au 5d)→ LUMO+2 (21% Au 6s, 19% Au 6p, 3% S 3p); 12.02% HOMO (15% Au 6s, 7% S 3p, 3% Au 5d) → LUMO+2; 10.30% HOMO–5 (14% S 3p, 8% Au 6s, 4% Au 5d) → LUMO+1 (19% Au 6p, 16% Au 6s, 4% S 3p)
			
B	2.92	0.4508	10.29% HOMO–15 (15% S 3p, 1% C 2p)→ LUMO (20% Au 6p, 12% Au 6s, 3% S 3p); 8.30% HOMO–13 (18% S 3p, 1% Au 6s, 1% Au 5d)→ LUMO+2
			
C	3.20	0.9101	9.43% HOMO–12 (20% S 3p, 1% Au 5d) → LUMO+3 (20% Au 6s, 11% Au 6p, 9% S 3p); 8.15% HOMO–3 (25% S 3p, 7% Au 5d, 3% C 2p) → LUMO+7 (22% Au 6s, 10% Au 6p, 1% S 3p)
			
D	3.44	1.3064	8.96% HOMO–9 (18% S 3p, 3% Au 6s, 1% Au 5d) → LUMO+6 (19% Au 6s, 18% Au 6p, 1% S 3p); 5.76% HOMO–9 → LUMO+7
			

**Table 3:** Principal Contributions in Terms of One-Electron Excited Configurations to the Main Spectral Features of Au_44_(SC_6_H_5_)_28_ Calculated at the PolTDDFT Level with the B3LYP XC Functional and TZP Basis Set and Using Fitted HDA

**excitation**	**excitation *E* (eV)**	***f***	**assignment**
A	1.78	0.1199	42.97% HOMO–1 (14% Au 6s, 13% S 3p, 3% Au 6p) → LUMO (15% Au 6p, 13% Au 6s); 35.09% HOMO (13% Au 6s, 11% S 3p, 2% Au 6p) → LUMO
			
B	2.18	0.1902	35.71% HOMO → LUMO+3 (21% Au 6s, 8% Au 6p, 5% S 3p); 19.25% HOMO–1 → LUMO+3
			
C	2.48	0.3526	41.99% HOMO–2 (15% S 3p, 5% Au 6s, 3% Au 5d) → LUMO+5 (16% Au 6s, 13% Au 6p, 2% S 3p); 12.62% HOMO–7 (28% S 3p, 9% Au 5d) → LUMO+3
			
D	3.0	0.7835	14.33% HOMO–4 (23% S 3p, 2% Au 5d) → LUMO+7 (22% Au 6s, 11% Au 6p); 7.57% HOMO–7 → LUMO+7
			
E	3.44	1.1661	12.60% HOMO–9 (18% S 3p, 4% Au 5d, 2% Au 6s) → LUMO+10 (13% Au 6p, 7% Au 6s, 3% S 3p); 10.55% HOMO–3 (19% S 3p, 8% Au 5d) → LUMO+12 (13% Au 6p, 8% Au 6s)
			

**Table 4:** Principal Contributions in Terms of One-Electron Excited Configurations to the Main Spectral Features of Au_52_(SC_6_H_5_)_32_ Calculated at the PolTDDFT Level with the B3LYP XC Functional and TZP Basis Set and Using Fitted HDA

**excitation**	**excitation *E*(eV)**	***f***	**assignment**
A	2.10	0.2127	17.92% HOMO–2 (13% S 3p, 6% Au 6s, 2% Au 6p)→ LUMO+2 (10% Au 6p, 7% Au 6s); 14.30% HOMO–4 (21% S 3p, 1% Au 5d) → LUMO+2
			
B	2.76	0.7238	6.10% HOMO–14 (18% S 3p, 3% Au 5d, 2% C 2p) → LUMO+4 (8% Au 6p, 6% Au 7s, 4% Au 6s)
			
C	3.24	1.1340	4.57% HOMO–46 (13% S 3p, 1% Au 5d, 1% Au 6s) → LUMO+1 (14% Au 6s, 9% Au 6p); 4.54% HOMO–22 (15% S 3p, 9% Au 5d, 8% Au 6s) → LUMO+6 (14% Au 6s, 13% Au 6p, 1% Au 7s)
			
D	3.62	1.3275	3.55% HOMO–9 (15% S 3p, 6% Au 5d, 3% Au 6s) → LUMO+13 (13% Au 6p, 12% Au 6s, 3% S 3p)
			
E	3.82	1.4371	3.85% HOMO–12 (15% S 3p) → LUMO+15 (10% Au 6s, 6% Au 6p)
			

**Figure 11:**
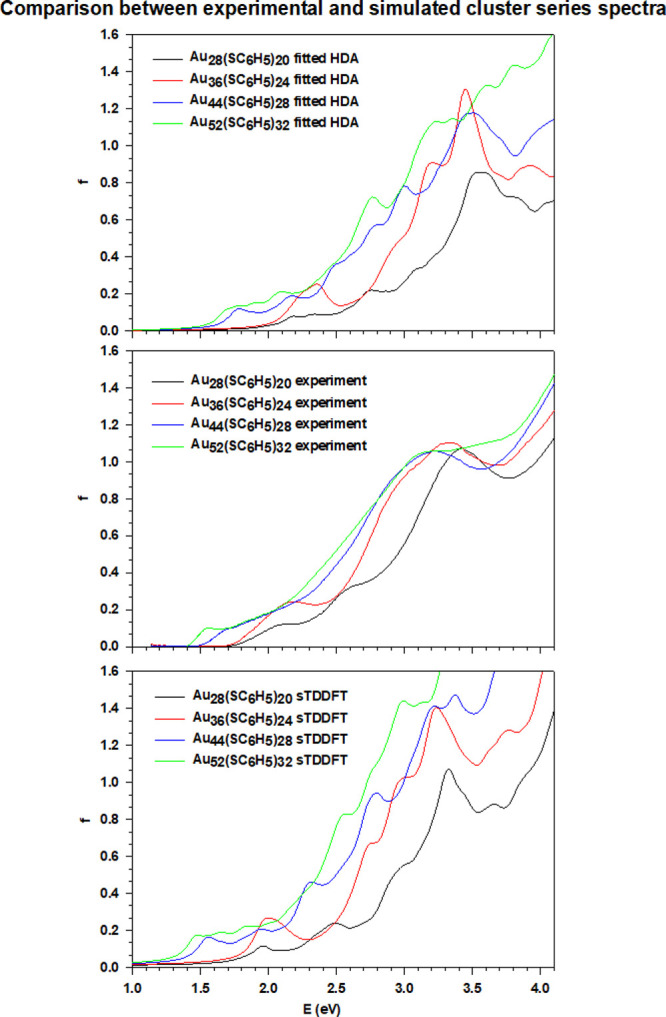
Comparison of the photoabsorption spectra for the cluster series: fitted HDA within the polTDDFT computational scheme (upper panel), experiment (central panel), and sTDDFT (lower panel).

It is interesting, at this point, to consider the cluster series as a whole and look at how the properties of the cluster evolve along it. The optical absorption spectra of the four clusters exhibit similar profiles (Figure 11), featuring an intense peak at ∼3.25 eV and less intense features moving to lower energy region. With increasing the cluster dimension, the density of the molecular orbitals near the HOMO and LUMO energy levels increases while the HOMO–LUMO energy gap decreases, with the exception of Au_36_(SC_6_H_5_)_32_. These observations provide an explanation for other two general features of the cluster series. First is the fact that the spectral profiles appear smoother and smoother moving to larger clusters, because far more features are present close to each other in energy. Second, the absorption intensities increase with energy and the cluster size. This is connected with the fact that the occupied molecular orbitals, due to their more pronounced ligand character, have a better overlap with the higher energy virtual molecular orbitals, since the lowest virtual ones have mainly metal character. Therefore, the associated transitions gain intensity going at higher energies.

Looking at Figure 11, it is possible to notice, with increasing cluster size, a redshift in the energy of the main absorption peaks. The same shift is more pronounced looking at the onset of the absorption profiles. Both the fitted HDA and sTDDFT simulations and experiment agree, at least qualitatively, about the order in which the absorptions for the different clusters begin: the lower absorption energy is that of Au_52_(SC_6_H_5_)_32_ followed by Au_44_(SC_6_H_5_)_28_ and, with a non-negligible energy difference, Au_36_(SC_6_H_5_)_24_ and Au_28_(SC_6_H_5_)_20_, which are practically overlapping. The onset of the absorption peak depends on the optical gap of the system, which is the energy gap plus the exciton binding energy, and indeed, it reflects the trend of the HOMO–LUMO energy difference obtained from the fitted HDA simulations. Such a similarity and uniform evolution in the UV–vis spectra of the “magic series” can be ascribed to the identical structure type (fcc) and protecting ligand used that make it similar to other, well-defined, quantum systems, like quantum dots or conjugated alkenes, with a clear quantum confinement nature.

It is worthwhile to note that besides these general trends, the present calculations are able to reproduce even minor details of the experiment. For example from Figure 11, in the experiment, the intensity profile of Au_36_(SC_6_H_5_)_24_ is always greater than that of Au_28_(SC_6_H_5_)_20_, but they touch each other around 2.6 eV. The theory reproduces fairly well this behavior, with a minimal shift of only 0.1 eV at higher energy. Another remarkable result is the behavior above 3.5 eV, where the three smaller clusters display a shallow minimum followed by an intensity increase, a feature not present in Au_52_(SC_6_H_5_)_32_; also, such effect is properly reproduced by theory.

In summary, the polTDDFT with B3LYP and fitted HDA has proven to be a very efficient and quantitative method to describe the optical properties of the investigated series. It must be also considered that the available experimental data have been all recorded at room temperature, so further experiment at low temperature^43^ would be extremely useful to better assess the performance of the present theoretical approach as well as to better understand the optical properties of these fascinating and intriguing systems. Although the topic of the present work is focused on the series of thiolate-protected gold metal clusters, the fitted HDA method is completely general and it is expected to keep its accuracy and computational efficiency in general. In fact, we compared the numerical and fitted HDA for a completely different system, namely, a decapeptide with explicit solvent consisting of 40 water molecules (GVGVP)_2_ (H2O)_40_, and obtained a very good match (see Figure S11 in the Supporting Information). This suggests that the conclusions of this work are general and are not restricted to the systems here considered.

## 5 Conclusions

In this work, we have implemented an RI technique to speed up the use of the hybrid xc functionals within the HDA approximation at the TDDFT theory level, solving the equations with the polTDDFT algorithm. In practice, the diagonal matrix elements of the nonlocal HF exchange kernel are calculated exploiting the RI instead of using the numerical integration as in the previous version of the code. The use of RI produced a speedup of almost a factor of 30 with respect to the previous numerical implementation, keeping the same level of accuracy, so that eventually the prediction of the optical photoabsorption spectrum via TDDFT/HDA requires essentially the same level of computational effort as a DFT SCF single-point calculation with a hybrid functional. Comparison with respect to the experimental photoabsorption spectra of the cluster series Au_8*n*+4_(SR)_4*n*+8_(*n* = 3*–6*; R = C_6_H_5_) demonstrated the high accuracy and efficiency of the method. The method has proven quantitatively accurate in both the high energy part of the spectrum, dominated by ligand contribution, and the lowest part of the spectrum, dominated by the metal-core absorption. Even minor spectral features and trends are well reproduced by theory. For comparison, we also employed sTDDFT and the much cheaper TDDFT+TB method to calculate the spectra, finding that it gives qualitatively correct results in the overall spectrum but with quantitative agreement with experiment only in the highest-energy part of the spectrum, dominated by the ligands, whereas the more accurate fitted HDA scheme performs much better with a predictive quantitative accuracy on the whole optical region. On the other hand, the sTDDFT method has proven to furnish absorption profiles with better agreement with respect to the experiment than the fitted HDA, although the discrepancy between the experimental maximum and the sTDDFT and fitted HDA is about 0.1 eV for both methods. Moreover, the sTDDFT is more computationally demanding than the fitted HDA. This finding suggests that the polTDDFT with the RI technique and hybrid xc functionals represents an accurate as well as computationally cheap approach, with optimal compromise between accuracy and computational efficiency, for applications to large metal clusters with sizes up to several hundreds of atoms.

## Data Availability

The data that support the findings of this study are available from the corresponding author upon reasonable request.

## Supporting Information Available

The ICM-OS plots, the induced density plots, the plots of occupied and virtual orbitals involved in the salient transitions, the photoabsorption profiles calculated by numerical and fitted HDA for (GVGVP)_2_ (H_2_O)_40_, and the geometries of all the gold clusters considered in this work (PDF)
