# Supplementary material for: A Resolution of Identity Technique to Speed up TDDFT with Hybrid Functionals: Implementation and Application to the Magic Cluster Series Au8n+4(SC6H5)4n+8 (n = 3–6)
Source: J Phys Chem A. 2023 Oct 31;127(44):9244–57. doi: 10.1021/acs.jpca.3c05368 (PMC10641850; doi:10.1021/acs.jpca.3c05368)
Supplement: Supplementary file 1 — jp3c05368_si_001.pdf [file jp3c05368_si_001.pdf]

SUPPORTING INFORMATION FOR:

A Resolution of Identity Technique to Speedup TDDFT with Hybrid Functionals: Implementation and Application to the Magic Cluster Series $Au_{8n+4}(SC_6H_5)_{4n+8}$ (n = 3-6)

Pierpaolo D'Antoni[1], Marco Medves[1], Daniele Toffoli[1], Alessandro Fortunelli[2], Mauro Stener[1]* and Lucas Visscher[3]*

[1]*Dipartimento di Scienze Chimiche e Farmaceutiche, Università di Trieste, Via Giorgieri 1, 34127 Trieste, Italy*

[2]*CNR-ICCOM, Consiglio Nazionale delle Ricerche, via Giuseppe Moruzzi 1, 56124 Pisa, Italy*

3 Department of Chemistry and Pharmaceutical Sciences, Vrije Universiteit Amsterdam, De Boelelaan 1083, Amsterdam, 1081 HV, The Netherlands

The ICM-OS and induced density plots do not show presence of any significant collective behaviour (Figure S1). Clear indication of it would be the presence of intense peaks in the ICM-OS plots far from the straight line corresponding to the excitation energy. Neither the induced density plots show any dipole shape (Figure S2)

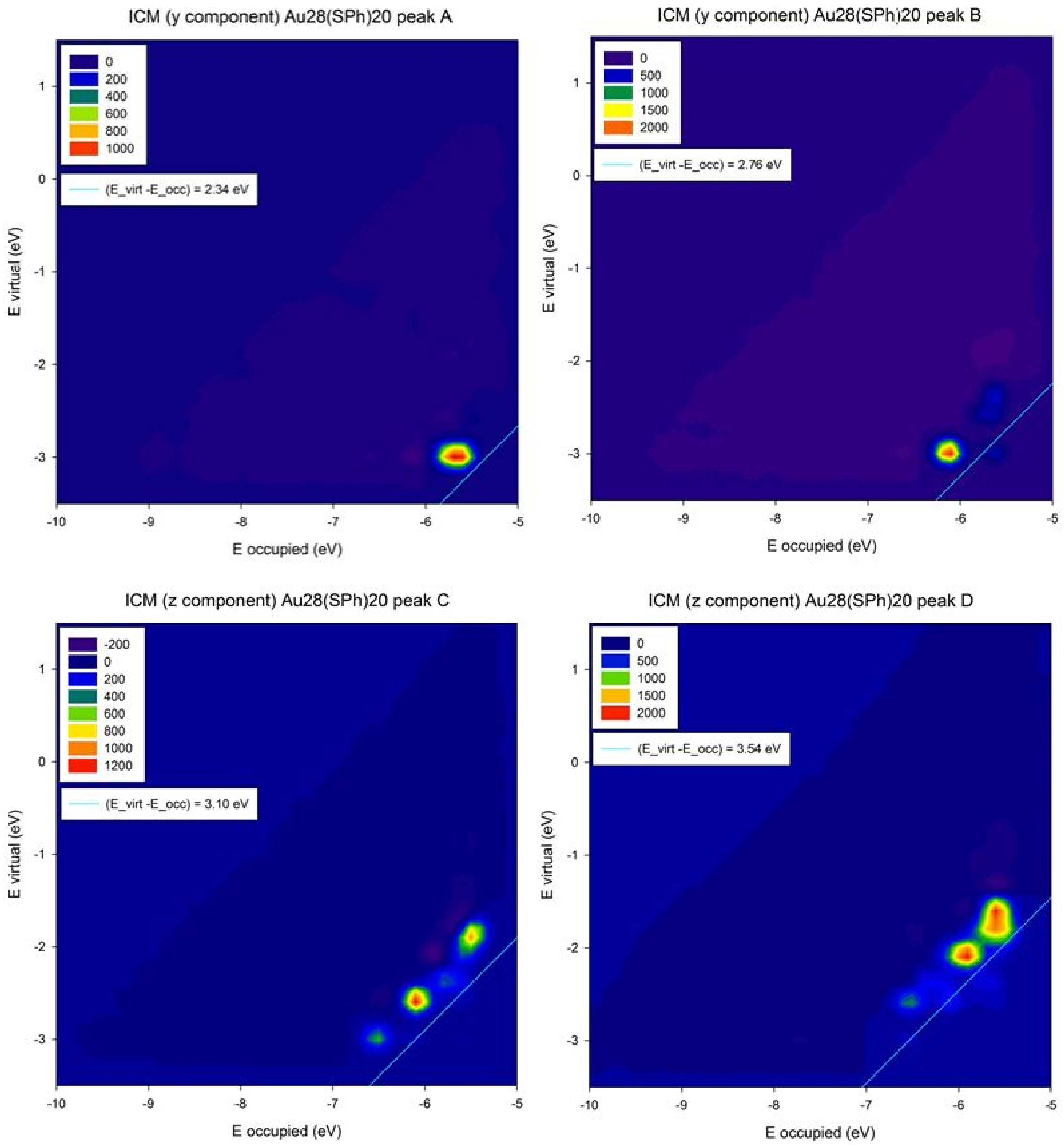

FIGURE S1: the ICM-OS plots for the most active component of the spectral features (A, B, C and D from figure 4 of the article) for $Au_{28}(SC_6H_5)_{20}$. The light blue line corresponds to pairs of occupied-virtual molecular orbitals with an energy difference equal to the excitation energy.

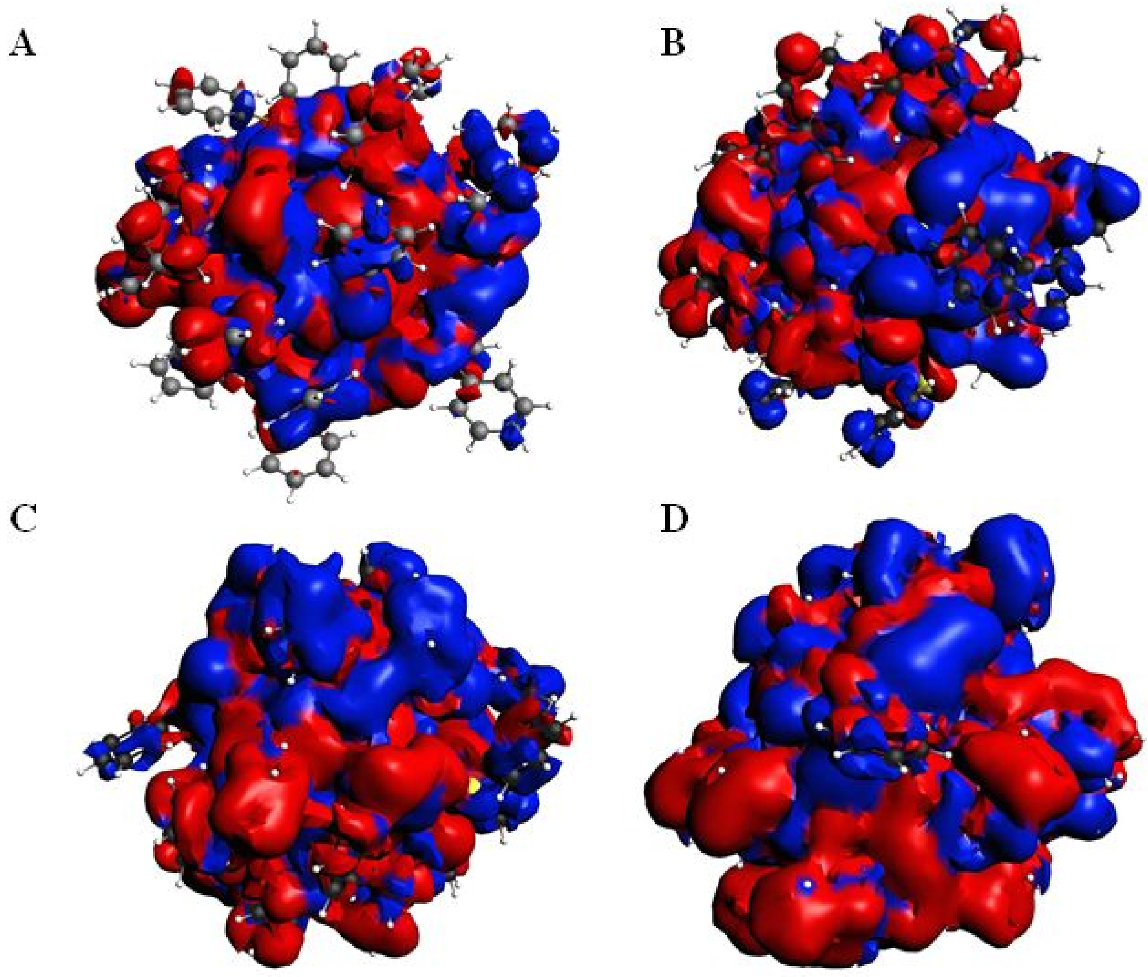

FIGURE S2: the induced density plots along the most active component of the spectral features (A, B, C and D from figure 4 of the article) for $Au_{28}(SC_6H_5)_{20}$.

In the following the occupied and virtual molecular orbitals, cited in the article for each cluster, starting from $Au_{28}(SC_6H_5)_{20}$ and moving up in cluster size, are reported. It is possible to appreciate how the occupied orbitals are more localized on the S-Au bonds with variable contribution from the ligand, on the other hand, the virtual orbitals closer to the LUMO energy are mainly localized at the metal core of the clusters.

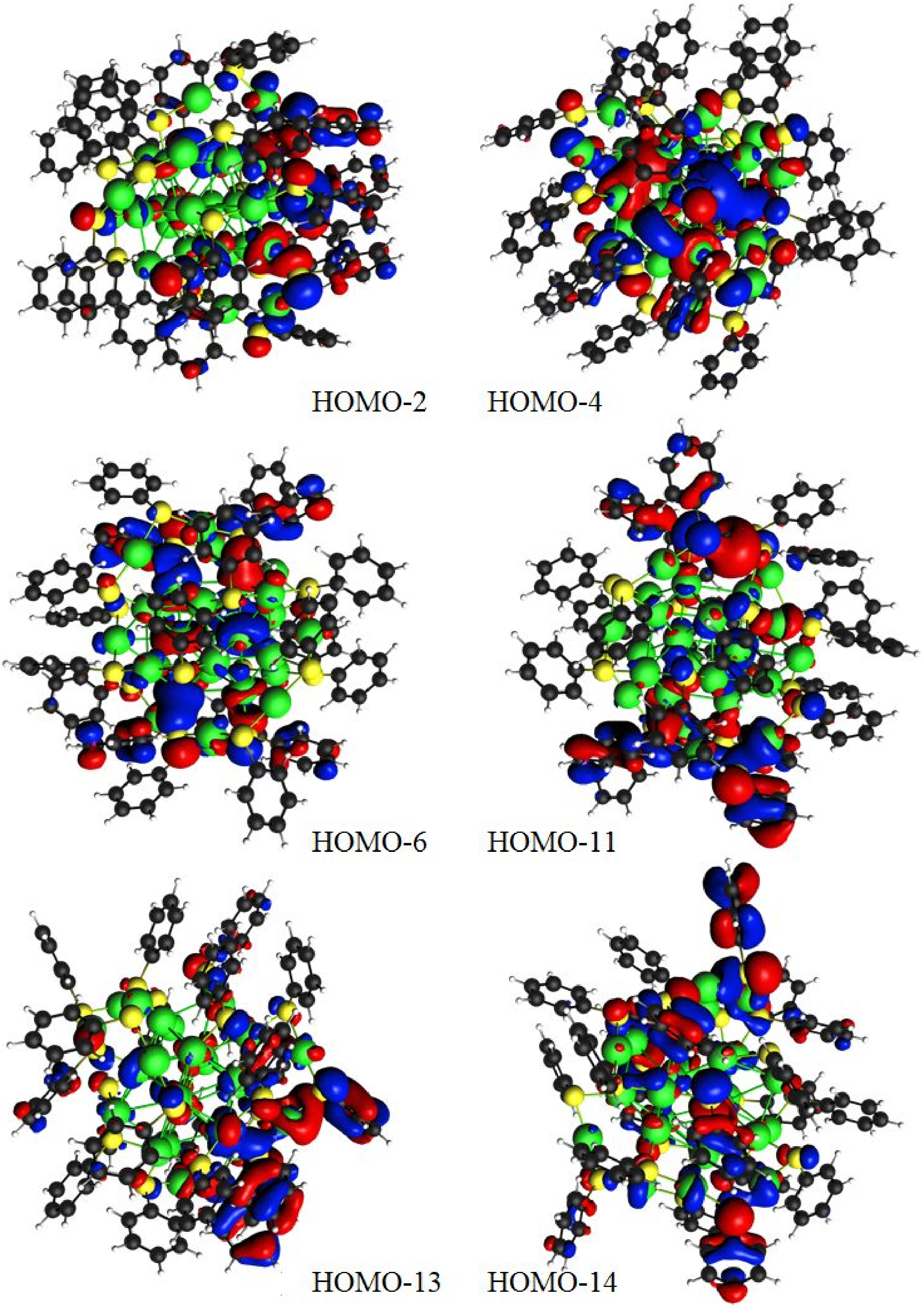

FIGURE S3: the occupied molecular orbitals of $Au_{28}(SC_6H_5)_{20}$ listed in Table 1 from the article.

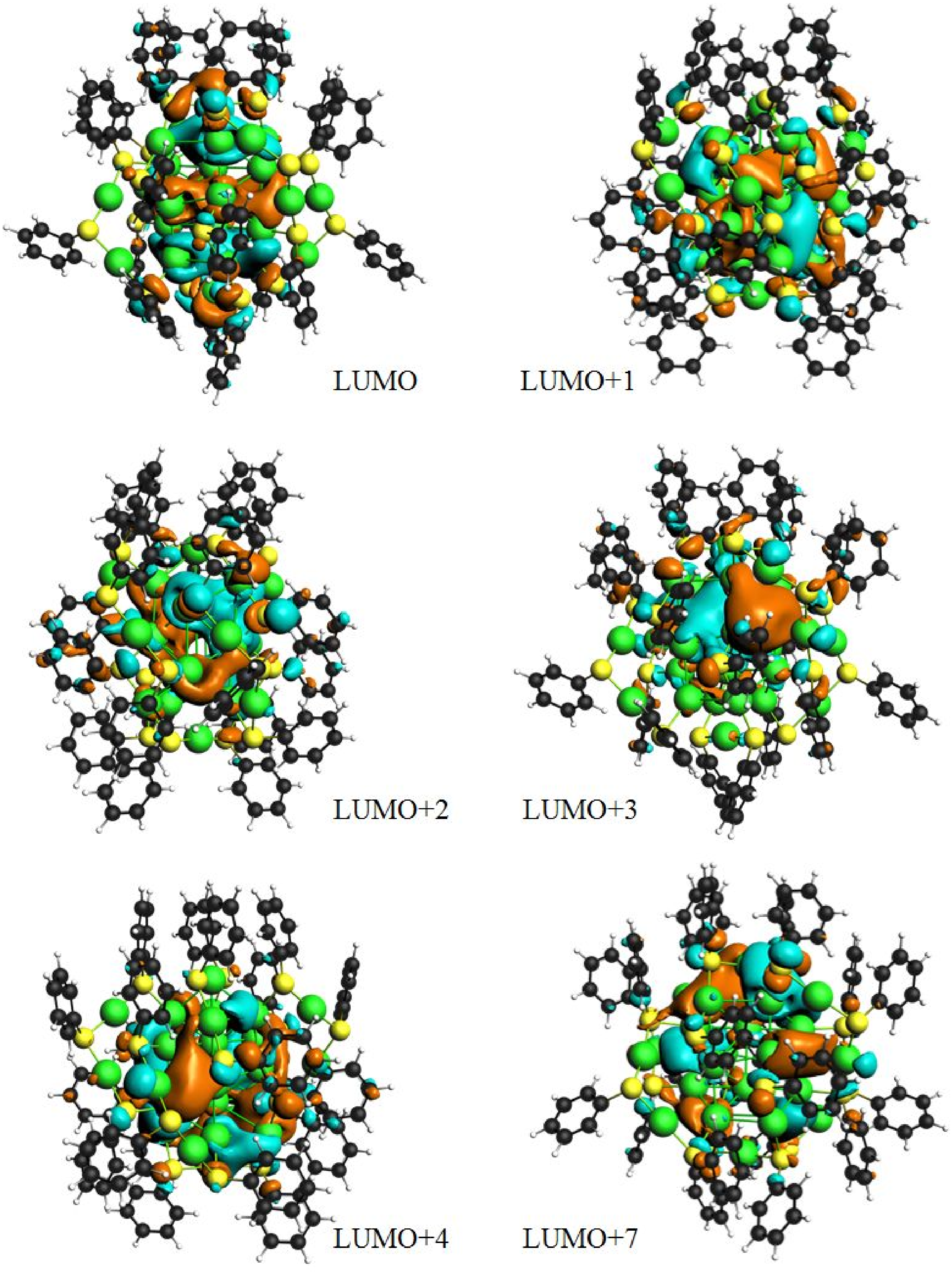

FIGURE S4: the virtual molecular orbitals of $Au_{28}(SC_6H_5)_{20}$ listed in Table 1 from the article.

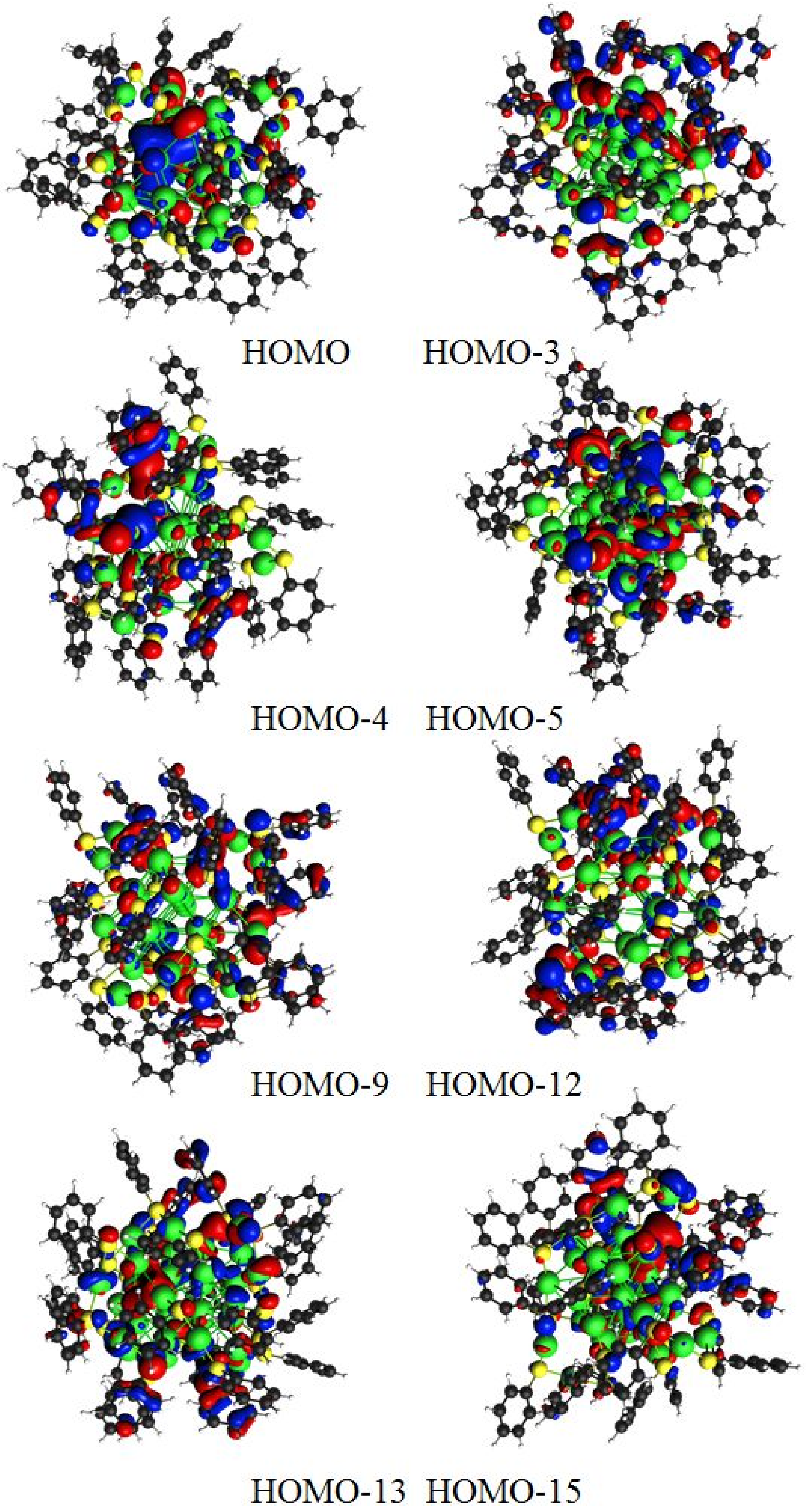

FIGURE S5: the occupied molecular orbitals of $Au_{36}(SC_6H_5)_{24}$ listed in Table 2 from the article.

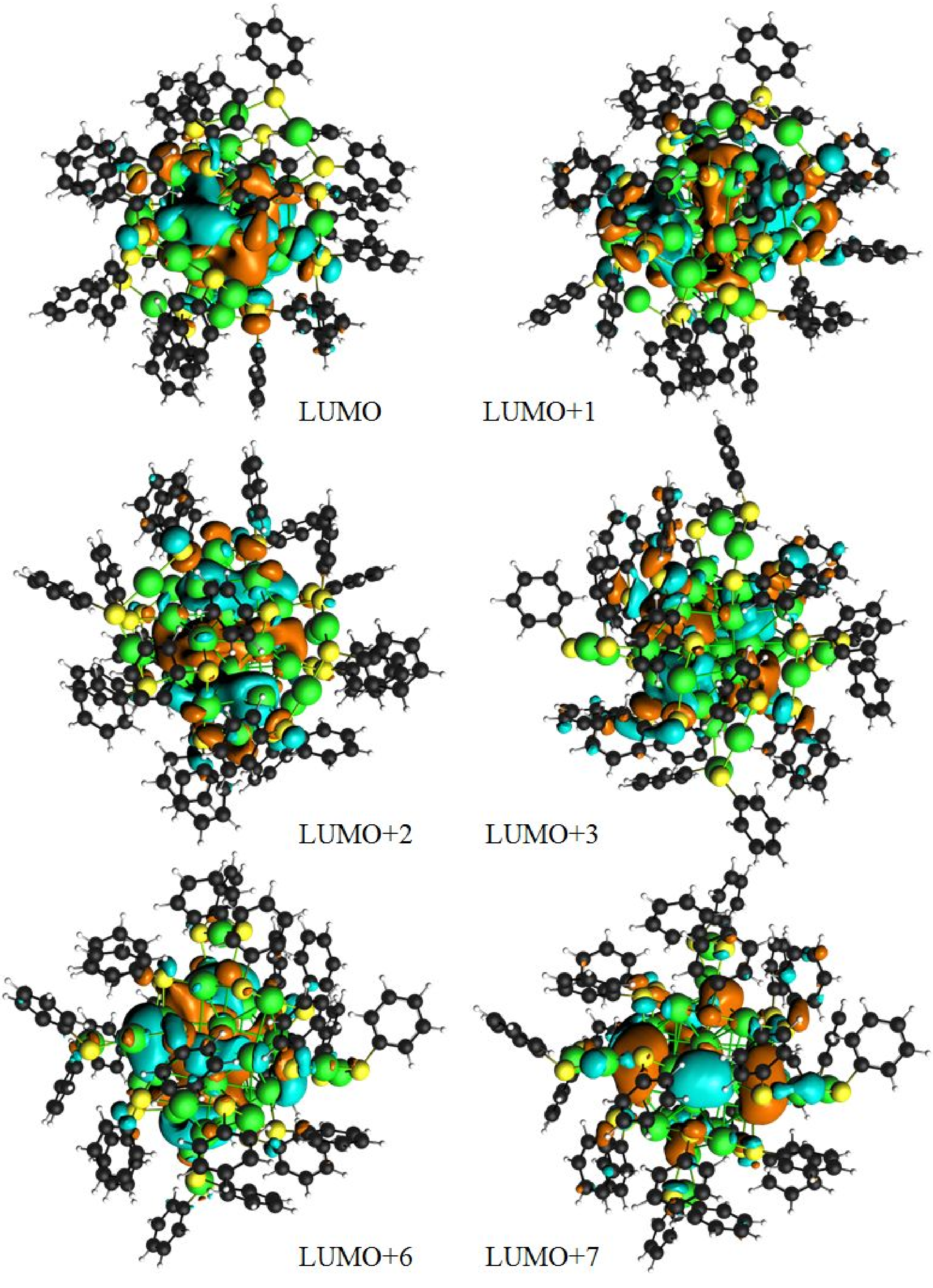

FIGURE S6: the virtual molecular orbitals of $Au_{36}(SC_6H_5)_{24}$ listed in Table 2 from the article.

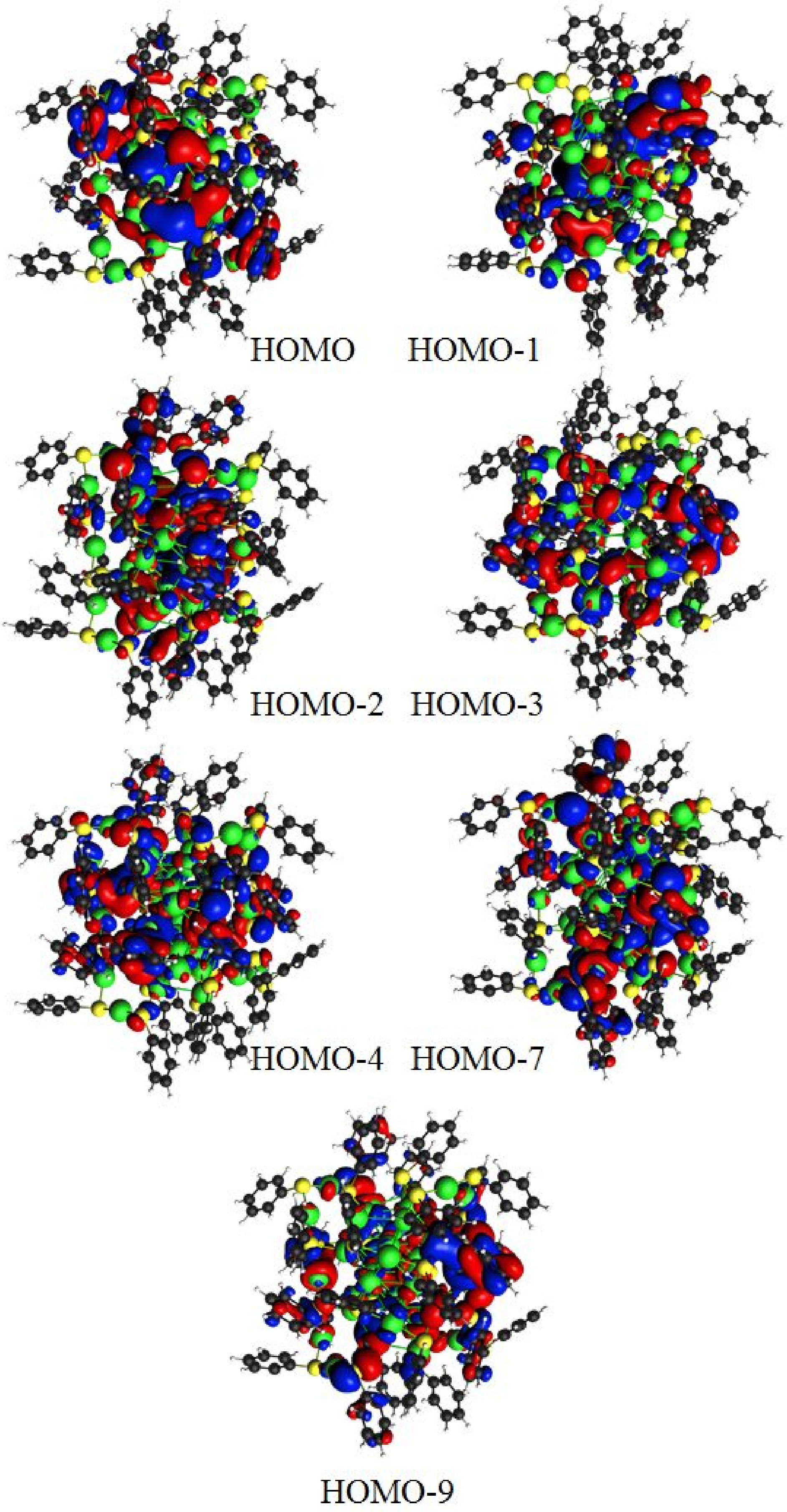

FIGURE S7: the occupied molecular orbitals of $Au_{44}(SC_6H_5)_{28}$ listed in Table 3 from the article.

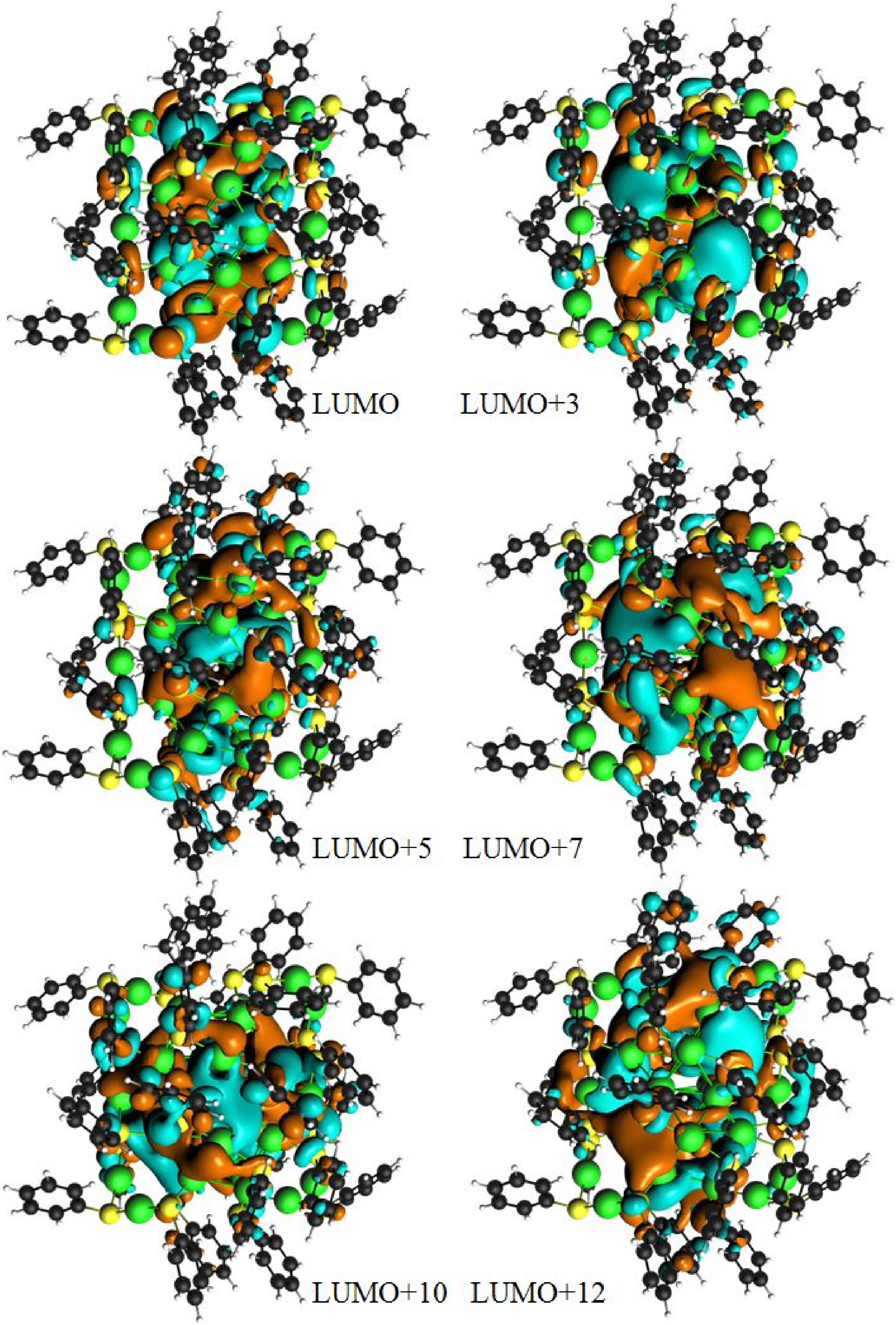

FIGURE S8: the virtual molecular orbitals of $Au_{44}(SC_6H_5)_{28}$ listed in Table 3 from the article.

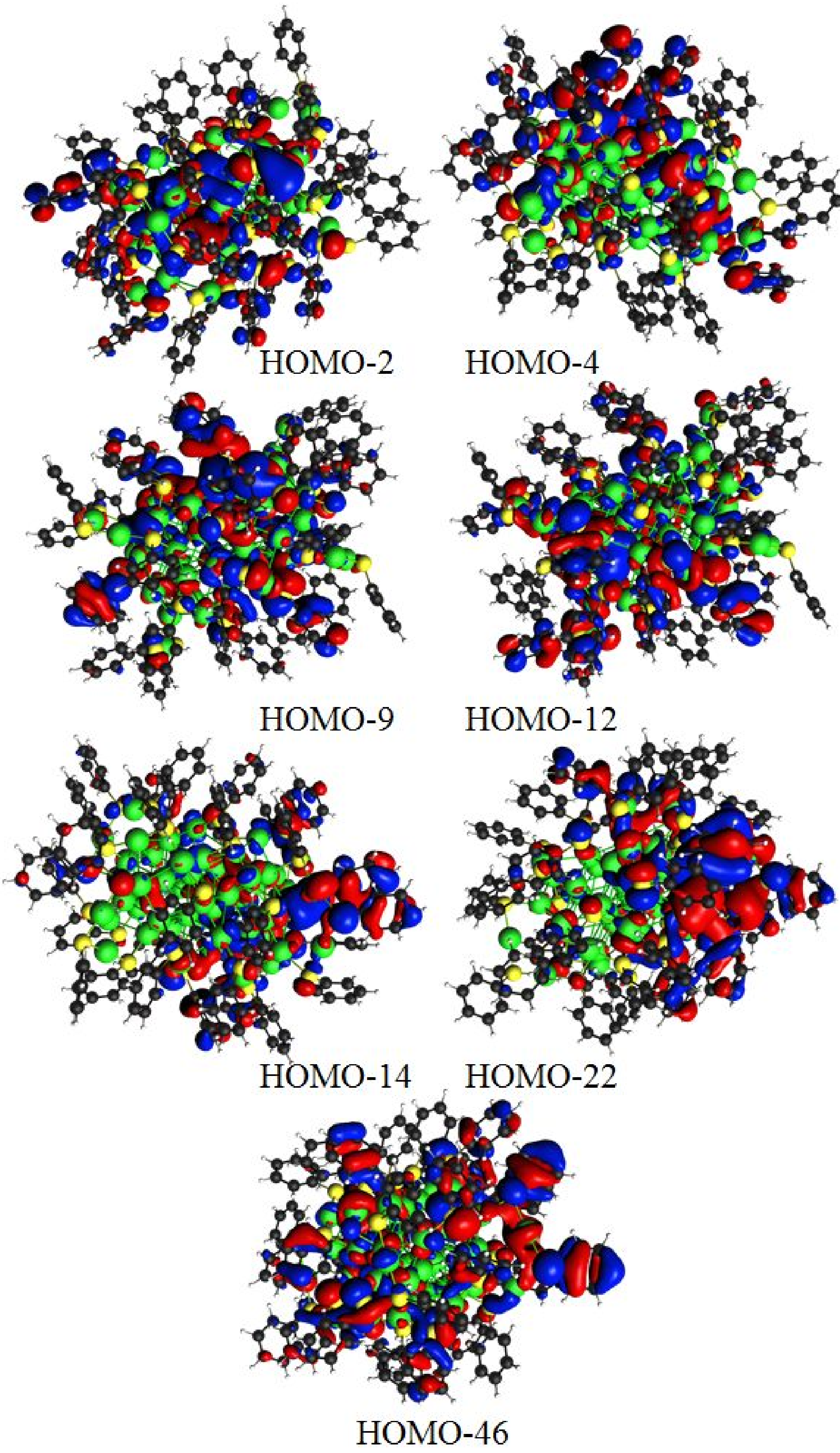

FIGURE S9: the occupied molecular orbitals of $Au_{52}(SC_6H_5)_{32}$ listed in Table 4 from the article.

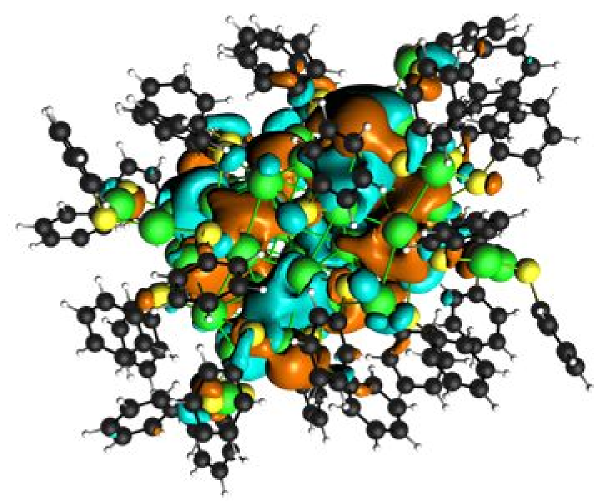

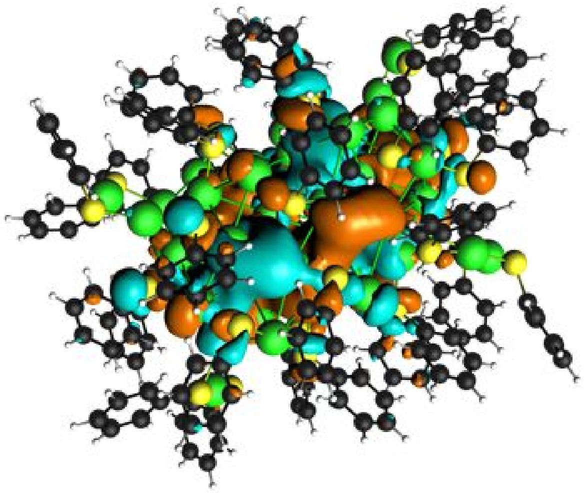

LUMO+1 LUMO+2

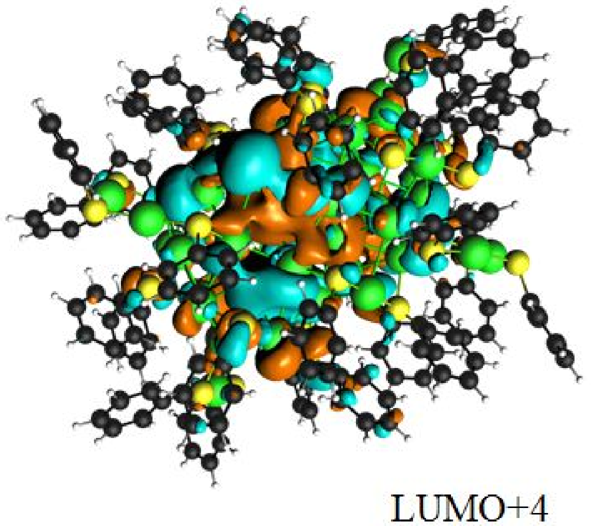

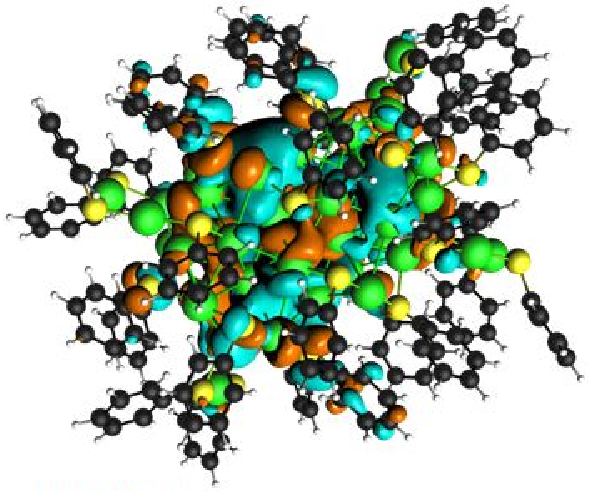

LUMO+4 LUMO+6

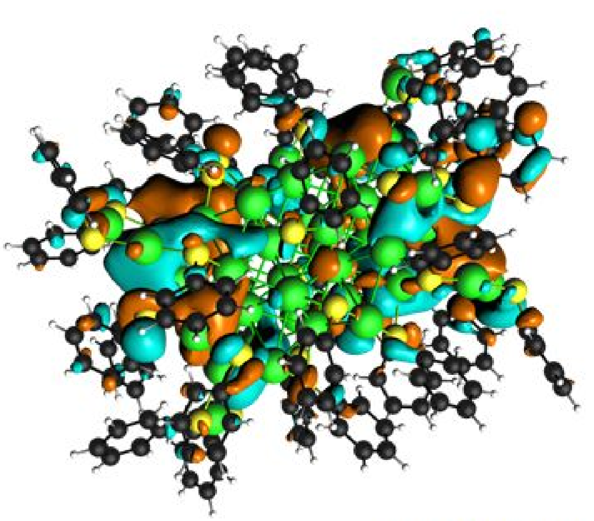

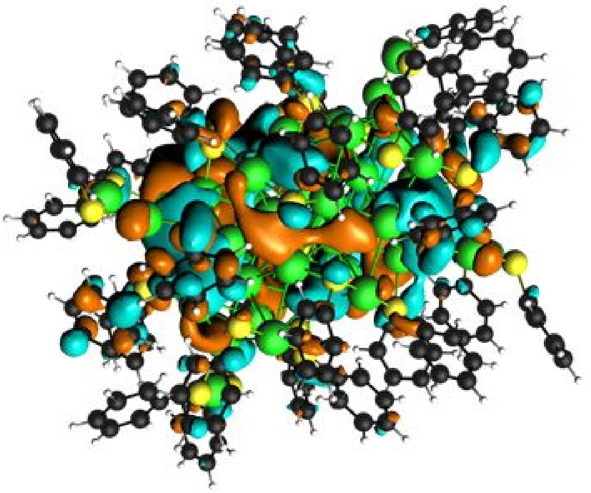

LUMO+13 LUMO+15

FIGURE S10: the virtual molecular orbitals of $Au_{52}(SC_6H_5)_{32}$ listed in Table 4 from the article.

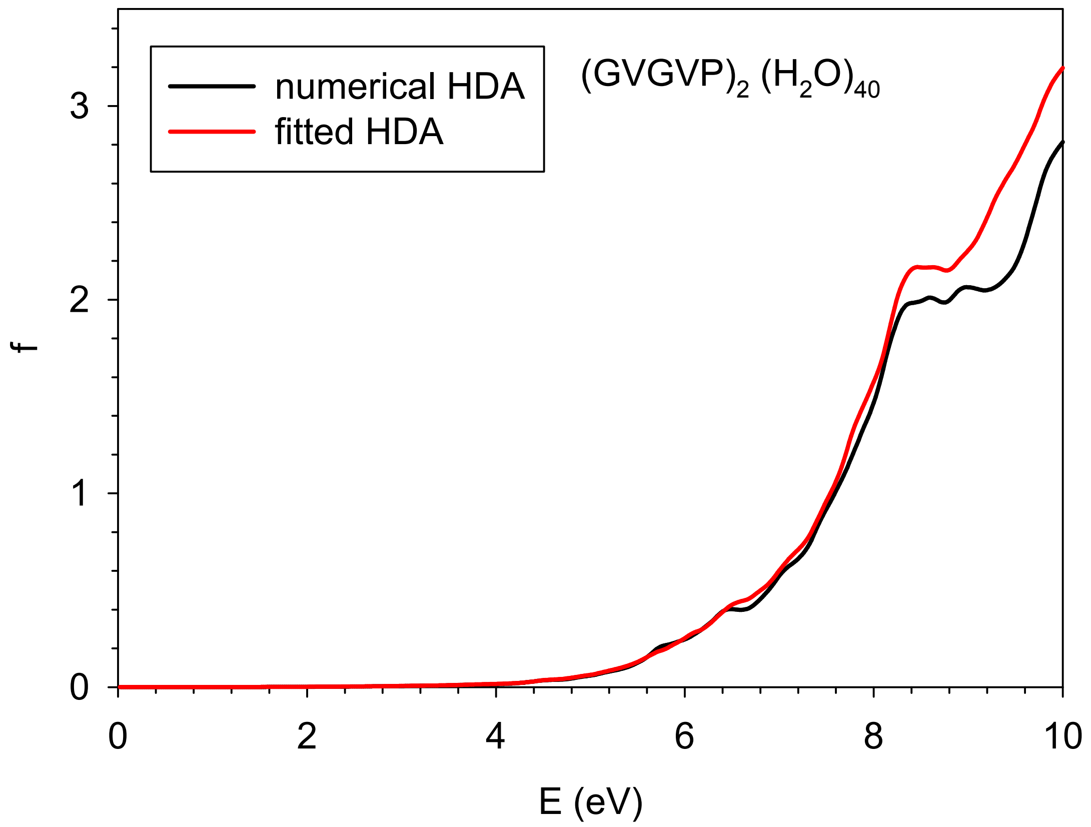

Figure S11. Numerical and fitted HDA for $(GVGVP)_2$ $(H_2O)_{40}$: a decapeptide with explicit solvent consisting of 40 water molecules.

Table S1. Coordinates of $Au_{28}(SC_6H_5)_{20}$ employed in the calculations.

Au	11.0050800000	13.1976800000	9.2972800000
Au	11.0294300000	15.9640600000	8.0186700000
Au	13.3403000000	14.4900900000	7.6959400000
Au	9.4903900000	11.1752200000	8.3701400000
Au	10.3679900000	14.3182200000	11.7430700000
Au	10.4031600000	17.1225000000	10.4088900000
Au	8.9791800000	17.7067500000	8.0764900000
Au	12.6216600000	15.1566900000	10.4699400000
Au	11.6480700000	14.8731100000	5.5938800000
Au	9.4238600000	13.5746200000	7.0475900000
Au	12.5294200000	12.6059100000	11.4584700000
Au	8.7126800000	15.2845700000	9.3360500000
Au	13.0873500000	17.0235100000	6.6483000000
Au	11.0340800000	19.0689600000	6.5584900000
Au	11.6804900000	12.0474400000	6.9022100000
Au	7.6508700000	16.4437100000	12.5345300000
Au	12.8629500000	18.3310100000	9.3176300000
Au	10.2925500000	12.0826000000	3.7240800000
Au	14.4035100000	12.8076900000	4.7675200000
Au	9.1942800000	16.4984000000	5.3612800000
Au	10.9872800000	10.2707800000	10.6821900000
Au	15.2042900000	16.3370000000	4.8159800000
Au	13.8389100000	11.5136800000	8.9206100000
Au	6.8705000000	18.6833000000	9.7951000000
Au	8.1724200000	12.4887800000	10.8414500000
Au	11.7624900000	16.7749000000	13.7546800000
Au	8.5509600000	9.8227500000	5.9159500000
Au	13.4441000000	13.6139600000	14.1003400000
S	6.0511600000	17.8035100000	11.7298200000
S	12.9679600000	11.0476200000	5.1978200000
S	12.7251300000	9.6898600000	9.4329600000
S	14.2966700000	16.7492900000	10.0725900000
S	7.7316500000	14.8881100000	6.0958900000
S	11.6000700000	13.9560000000	3.4372200000
S	10.4553400000	18.2002800000	12.4889800000
S	6.7759200000	14.1458900000	10.0984400000
S	14.5056400000	18.4738700000	5.4078000000
S	15.2379200000	13.1255400000	8.0890900000
S	9.2074500000	10.8058100000	12.0431800000
S	10.4550300000	18.2100200000	4.4742000000
S	12.8986400000	15.4807000000	15.2833400000
S	16.0239500000	14.3033000000	4.2416000000
S	7.5797300000	19.5477000000	7.7692700000
S	9.0688500000	14.9699300000	13.6261100000
S	11.5843500000	20.0823700000	8.5801200000
S	8.0226700000	9.3299900000	8.1181600000
S	9.1303600000	10.1370600000	3.7150400000
S	13.9713600000	11.6138300000	13.0833900000
C	5.9231639360	19.2202754367	12.8384844063
C	4.9911186720	20.2209783788	12.5179980769
C	4.8057063470	21.3031344153	13.3804310945
C	5.5401926845	21.3958469971	14.5683099377
C	6.4702436376	20.4015326648	14.8829517600
C	6.6675996242	19.3168618196	14.0224425262
C	11.6835565642	14.8531925269	16.4531702162
C	10.5395918160	15.5999617760	16.7724819139
C	9.6463524014	15.1282295674	17.7378150926
C	9.8820523390	13.9154575423	18.3920412046

C	11.0222036493	13.1712320659	18.0729736728
C	11.9226256359	13.6349835967	17.1110977067
C	13.0282375430	10.3634379528	13.9538912440
C	13.6153141066	9.0925937741	14.0478119382
C	12.9555568740	8.0668121418	14.7297861689
C	11.7110693822	8.2999483064	15.3222280426
C	11.1219938002	9.5647647223	15.2136386517
C	11.7701324638	10.5948524376	14.5299260084
C	13.9497199874	9.7290893089	5.9121847999
C	13.2868684310	8.5260784196	6.1976867729
C	13.9950961333	7.4541437717	6.7434966133
C	15.3649245244	7.5656899281	7.0001764198
C	16.0274663228	8.7600772748	6.7009585896
C	15.3264206766	9.8419356348	6.1619884140
C	8.0836087653	13.5956680577	14.2184512307
C	6.7610854159	13.3510420152	13.8159631079
C	6.0772596891	12.2386894448	14.3137015918
C	6.6965537776	11.3720216270	15.2196100371
C	7.9985165396	11.6432721211	15.6503264873
C	8.6949148203	12.7471336956	15.1534884099
C	10.3029035950	8.7908126921	3.4658928639
C	9.9389842728	7.4941011447	3.8696932270
C	10.8059145309	6.4215844700	3.6489336970
C	12.0384016021	6.6272044726	3.0198867767
C	12.3927753077	7.9139251735	2.6051761416
C	11.5315938055	8.9936236144	2.8208546711
C	15.9779247879	14.1802456985	2.4412204800
C	16.8620477823	13.2770316545	1.8332140912
C	16.8173401055	13.0865458420	0.4487468117
C	15.8987787864	13.7939863307	-0.3337098319
C	15.0275753278	14.7038967412	0.2748166402
C	15.0666062917	14.8994924150	1.6565919272
C	15.7711797478	19.0093351915	6.5761722536
C	16.8413739163	18.1884383748	6.9743601634
C	17.8016019455	18.6607100211	7.8691352602
C	17.7093606514	19.9565166704	8.3877722593
C	16.6531203963	20.7775567558	7.9868170173
C	15.6895715997	20.3139986441	7.0845624406
C	12.6436582468	21.4792101854	8.2384137741
C	13.4807678667	21.9529181664	9.2593435696
C	14.2328638837	23.1109137551	9.0581102862
C	14.1677710083	23.7970799441	7.8404574899
C	13.3271455705	23.3263872547	6.8261344117
C	12.5583066415	22.1765437718	7.0238695431
C	9.2839627959	19.3264462014	3.7016455523
C	8.4975346168	18.8341469350	2.6472483140
C	7.6717430836	19.7020097353	1.9317335491
C	7.6255691592	21.0627449571	2.2548513977
C	8.4067290111	21.5514449451	3.3063112238
C	9.2348580805	20.6886165497	4.0307915373
C	11.2469229641	19.7975450373	12.3918027986
C	12.5450947107	20.0475612282	12.8609029276
C	13.0338698923	21.3576839104	12.8768229944
C	12.2440613994	22.4153038817	12.4145916234
C	10.9617756550	22.1575105517	11.9166592256
C	10.4595983478	20.8553279324	11.9056770215
C	10.9406814114	15.0871742357	2.2284240082
C	9.8693312784	14.7615537984	1.3843599104
C	9.4892738314	15.6487871942	0.3721677778
C	10.1734284247	16.8551224151	0.1875396307

C	11.2418957737	17.1785646580	1.0312214514
C	11.6236437782	16.3029249286	2.0485626435
C	16.1972398561	13.8276791600	9.4264031796
C	16.9489210859	14.9803825737	9.1473848865
C	17.7599796955	15.5404116728	10.1365428966
C	17.8292616933	14.9617672856	11.4072544175
C	17.0824667944	13.8124693181	11.6842720584
C	16.2713908007	13.2432833363	10.6991797081
C	15.0218136856	17.4347920026	11.5552294279
C	15.0959071125	16.6828201336	12.7332221294
C	15.7486689795	17.2022303179	13.8542328635
C	16.3452241587	18.4657024690	13.8004740912
C	16.2983781670	19.2024084234	12.6097010070
C	15.6414702175	18.6947195317	11.4876829418
C	5.7552268583	13.5013169303	8.7831792894
C	4.8934642820	14.4019058036	8.1362413628
C	3.9842575499	13.9367171293	7.1838112578
C	3.9211744390	12.5749270427	6.8709618362
C	4.7762573062	11.6773460091	7.5184794640
C	5.6916028125	12.1352979987	8.4695823933
C	8.9449721264	7.8378402925	8.4980033558
C	8.6293583775	7.1885094524	9.7011043059
C	9.3002478167	6.0164687801	10.0604256443
C	10.2694308899	5.4667754261	9.2165822718
C	10.5642868315	6.1006763498	8.0045127450
C	9.9161460168	7.2844143318	7.6485743677
C	13.7375044393	8.5869194036	10.4162380916
C	13.1493564116	7.3887226913	10.8539593993
C	13.9192347350	6.4497232240	11.5407964455
C	15.2757016753	6.6912154938	11.7901603028
C	15.8593527904	7.8817601195	11.3454335336
C	15.0958859816	8.8313059961	10.6596659782
C	6.3983947714	19.0330123765	6.5194700090
C	5.8259845435	17.7527351106	6.4810005965
C	4.9845624093	17.3948477899	5.4268500386
C	4.6938201481	18.3124132545	4.4106277435
C	5.2516282522	19.5934415522	4.4590031431
C	6.1041800948	19.9560517716	5.5054228959
C	7.1594926757	14.0119530470	4.6363358724
C	7.0809537296	14.6333001963	3.3818786223
C	6.5446656881	13.9390505477	2.2934973580
C	6.0734925783	12.6319024932	2.4465121113
C	6.1438539655	12.0172688939	3.7014908783
C	6.6858545701	12.7002196544	4.7908959704
C	8.0929057134	9.4322528520	12.2550307872
C	6.7346567313	9.5121872587	11.9183277807
C	5.8862558483	8.4348945985	12.1936985105
C	6.3839667164	7.2811724419	12.8081979900
C	7.7377806194	7.2126849084	13.1589529108
C	8.5928265717	8.2820936183	12.8891689009
H	4.4221616565	20.1521072854	11.5927296849
H	4.0838804534	22.0749143849	13.1222811461
H	5.3906492458	22.2382614471	15.2398705077
H	7.0519925172	20.4663122915	15.8000237086
H	7.4020895788	18.5482468528	14.2600844261
H	10.3420438997	16.5328422264	16.2479094775
H	8.7549213987	15.7085521197	17.9658003657
H	9.1810579939	13.5507979235	19.1393870540
H	11.2152518253	12.2200453230	18.5643089325
H	12.8018714604	13.0451511471	16.8564722241

H	14.5783979369	8.9072042563	13.5787928856
H	13.4216725528	7.0855264540	14.7891277015
H	11.2019181233	7.5027005017	15.8593003405
H	10.1421592998	9.7516471204	15.6473645523
H	11.2965121082	11.5688402097	14.4264817133
H	12.2214771927	8.4394409879	5.9976480618
H	13.4681904934	6.5322643861	6.9793021768
H	15.9091783546	6.7301917520	7.4333482176
H	17.0939479219	8.8578760510	6.8940448094
H	15.8375964153	10.7776964102	5.9412748733
H	6.2767438436	14.0287980711	13.1153325060
H	5.0590336378	12.0472550786	13.9817366916
H	6.1695301694	10.4945473534	15.5869540613
H	8.4781339401	10.9970421920	16.3825000645
H	9.7046969503	12.9600120572	15.4983524891
H	8.9842448487	7.3312793795	4.3678037261
H	10.5138222281	5.4254972234	3.9749378344
H	12.7174589584	5.7932616041	2.8580952277
H	13.3517062523	8.0892601863	2.1224108091
H	11.8213630983	9.9964678323	2.5139062339
H	17.5683134368	12.7188020056	2.4437205303
H	17.5001245217	12.3782899160	-0.0153536465
H	15.8615180179	13.6369706515	-1.4104567219
H	14.3043480138	15.2580350362	-0.3201066378
H	14.3781299508	15.5929326968	2.1328790156
H	16.9221092435	17.1811361006	6.5683420931
H	18.6289604579	18.0129756718	8.1524857320
H	18.4555959592	20.3230824210	9.0890907815
H	16.5642230720	21.7894506854	8.3738188067
H	14.8743737117	20.9654090542	6.7834941474
H	13.5381456558	21.4132400648	10.2018822309
H	14.8790758711	23.4701150868	9.8569857646
H	14.7693786802	24.6885422262	7.6822812971
H	13.2739337493	23.8496655224	5.8737684797
H	11.9076805629	21.8020152243	6.2347342600
H	8.5485710088	17.7794848746	2.3847869585
H	7.0615808662	19.3095662308	1.1208289671
H	6.9797126317	21.7350827370	1.6948524590
H	8.3704734099	22.6070113598	3.5685712612
H	9.8373308206	21.0625340038	4.8574370919
H	13.1617491110	19.2249946510	13.2199766735
H	14.0395088617	21.5438534427	13.2487568285
H	12.6279508183	23.4329765100	12.4252332055
H	10.3458742280	22.9701926698	11.5380338070
H	9.4557149053	20.6513945158	11.5357363216
H	9.3385029857	13.8226730266	1.5283938737
H	8.6521471491	15.3923819308	-0.2744266891
H	9.8700301651	17.5443967077	-0.5973227681
H	11.7727668562	18.1208362304	0.9119911625
H	12.4399218501	16.5637736123	2.7202153246
H	16.8731354227	15.4479695456	8.1679101388
H	18.3167025340	16.4488100658	9.9183210384
H	18.4462869780	15.4157190716	12.1793116913
H	17.1160003534	13.3595580671	12.6731231917
H	15.6879294671	12.3510818225	10.9193993794
H	14.6576738317	15.6848343849	12.7594868090
H	15.7790404530	16.6145481385	14.7696735926
H	16.8470254665	18.8710247310	14.6758814445
H	16.7749976664	20.1793307038	12.5531768784
H	15.6077163202	19.2658561332	10.5604847184

H	4.9299450597	15.4587513791	8.3931724673
H	3.3220602469	14.6453278289	6.6901649680
H	3.2159376113	12.2196335563	6.1236699900
H	4.7430567653	10.6155631051	7.2809743712
H	6.3577578708	11.4355937353	8.9711369549
H	7.8621351552	7.5982402890	10.3508428789
H	9.0533328688	5.5358740107	11.0049436759
H	10.7841389998	4.5512251016	9.4964444288
H	11.3008972373	5.6737221798	7.3264584476
H	10.1495154089	7.7730251846	6.7048725468
H	12.0961446195	7.1969315035	10.6530610748
H	13.4562407598	5.5250253691	11.8795374644
H	15.8731685857	5.9574506002	12.3256791247
H	16.9123848000	8.0798921659	11.5361784240
H	15.5472119361	9.7578415309	10.3080574836
H	6.0691512961	17.0320977001	7.2579452705
H	4.5735829456	16.3872158036	5.3935002313
H	4.0448970082	18.0298180557	3.5849543044
H	5.0435492280	20.3143230959	3.6718666427
H	6.5600802054	20.9425280021	5.5243402259
H	7.4557325516	15.6478534825	3.2587209301
H	6.5066883280	14.4232515758	1.3193149470
H	5.6619136477	12.0935459449	1.5958653435
H	5.7906093694	10.9971249865	3.8387050983
H	6.7440290700	12.2196243522	5.7650051845
H	6.3502647106	10.4149875132	11.4445212656
H	4.8361041256	8.4968954007	11.9156846719
H	5.7243679190	6.4397614381	13.0092428143
H	8.1355974545	6.3189349659	13.6355774362
H	9.6469430772	8.2289803900	13.1559633625

Table S2. Coordinates of $Au_{36}(SC_6H_5)_{24}$ employed in the calculations.

Au	11.3668570000	0.7126530000	0.8836020000
Au	14.8793210000	0.2294470000	1.3684400000
Au	11.1334170000	2.0157630000	3.6093460000
Au	13.6926870000	2.9332410000	3.2409780000
Au	16.7358810000	4.0543410000	2.6778920000
Au	16.0945780000	1.7503220000	4.5904580000
Au	13.2798260000	0.6105310000	4.6088160000
Au	15.8882570000	-1.1854960000	5.7837420000
Au	17.4772960000	0.2963820000	8.4983510000
Au	14.7023700000	-0.6861890000	8.3860950000
Au	12.6817380000	-2.4000480000	9.7840280000
Au	12.3494760000	0.3876030000	7.3667620000
Au	14.8477400000	1.6883800000	7.0661090000
Au	17.3664910000	2.8332390000	6.8154140000
Au	18.2432100000	5.5554480000	5.4662350000
Au	15.2951390000	4.2052820000	5.5866170000
Au	12.5055390000	3.0353910000	5.7921690000
Au	9.5887190000	1.6697130000	6.1280340000
Au	10.1032020000	4.6447970000	4.3150840000
Au	13.2573870000	5.6209550000	4.3195990000
Au	15.8589300000	6.5204840000	4.2013240000
Au	14.8721610000	9.3681520000	3.9888500000
Au	11.8257420000	8.8574440000	5.6386820000
Au	11.6855250000	5.4655500000	6.9060010000
Au	14.4243160000	6.6989490000	6.5499720000
Au	16.4265120000	5.3588060000	8.2657130000
Au	13.8227660000	4.2128600000	8.1618840000
Au	11.2808340000	3.0764100000	8.2756440000
Au	10.2122340000	0.1620070000	10.0076370000
Au	13.5076910000	1.4945230000	9.5649330000
Au	15.9898770000	3.0084320000	9.5435650000
Au	19.3811840000	3.8672120000	9.5706510000
Au	12.6487280000	7.0686600000	9.3160440000
Au	14.7746020000	5.3197650000	10.4563600000
Au	12.2077650000	4.1640780000	11.1572830000
Au	17.2882390000	5.3059000000	12.1543450000
S	13.2161350000	-0.1204250000	-0.2046490000
S	16.6406890000	0.2179030000	2.8500380000
S	9.4836510000	1.4701820000	1.9652320000
S	14.9344080000	3.5019960000	1.2910940000
S	18.8464660000	4.3356630000	3.6144630000
S	13.8112110000	-1.6754270000	4.9627380000
S	18.0087120000	-0.7476640000	6.5126530000
S	11.5004250000	5.5663520000	2.7206280000
S	16.5285110000	8.3061590000	2.7537330000
S	19.6252860000	3.2749210000	7.3613450000
S	14.9537690000	-2.7582980000	9.5131690000
S	10.4265470000	-2.1311600000	9.9766390000
S	10.1926730000	-0.4620150000	6.8166170000
S	8.6486740000	3.7749290000	5.8987060000
S	10.2738660000	7.2754290000	6.2387850000
S	13.3480920000	10.5809760000	5.2335970000
S	9.7447840000	2.4031840000	9.9736290000
S	10.9569860000	5.9751150000	10.4732140000
S	13.9298940000	8.5126770000	8.0294640000
S	17.8971740000	6.9219800000	7.3101830000
S	17.4833980000	1.4317810000	10.5002990000
S	13.4416320000	2.3052050000	11.7793560000

S	15.2598700000	6.3669910000	12.5497990000
S	19.3093970000	4.2132060000	11.8455660000
C	12.9482773539	0.2101430108	-2.8918325711
C	13.4748951289	0.7917292448	-1.7260415546
C	14.2241028603	1.9687816151	-1.8081482696
C	14.4304403312	2.5763453714	-3.0490610373
C	13.9089528667	2.0027748558	-4.2141742307
C	13.1700591658	0.8160239384	-4.1328101214
C	18.0880234594	0.8671998681	2.0280972637
C	19.3003875409	0.7786322387	2.7318834837
C	20.4715261918	1.2829319306	2.1646970869
C	20.4508915325	1.8685618965	0.8939206615
C	19.2468051105	1.9365679760	0.1868751122
C	18.0655213608	1.4417845917	0.7477344185
C	9.6786119363	3.7418619634	0.3099567091
C	9.1474848312	4.9049652859	-0.2481343399
C	7.8528276373	5.3212343632	0.0783310531
C	7.0931726168	4.5657417122	0.9757485861
C	7.6198982221	3.4051997617	1.5490614824
C	8.9174714865	2.9870410628	1.2128683959
C	15.2179872968	6.1534762082	0.4419879356
C	14.8181052851	7.2705091117	-0.2947067413
C	13.6161364753	7.2503155788	-1.0086167970
C	12.8143987511	6.1050636500	-0.9750481221
C	13.2000291726	4.9906538254	-0.2269105257
C	14.4074163765	5.0102682894	0.4883565418
C	20.9073948716	6.0687688349	3.0499452209
C	19.7687505384	5.4279571979	2.5359403788
C	19.4613427515	5.5425543910	1.1722226504
C	20.2917586345	6.2891820138	0.3316999837
C	21.4322491724	6.9187548184	0.8403262505
C	21.7336409010	6.8078017255	2.2023001599
C	12.9358788879	-2.4446694678	2.4806104082
C	14.0047762763	-2.4963924447	3.3878443944
C	15.1333978815	-3.2774285770	3.0982798389
C	15.1928174034	-3.9880285656	1.8966419839
C	14.1282842305	-3.9395671219	0.9901515316
C	12.9990820543	-3.1702163585	1.2900889800
C	19.1748223646	-3.1007986365	5.7533447834
C	18.8637227624	-2.2806238638	6.8496667231
C	19.3408212777	-2.6098069244	8.1250294225
C	20.1159851424	-3.7587164892	8.3038852081
C	20.4262525119	-4.5792587343	7.2140329211
C	19.9540845946	-4.2440455563	5.9393150695
C	11.9125332751	8.1673008381	1.9324860620
C	10.9579100872	7.2242231855	2.3440439559
C	9.5918984666	7.5378689144	2.2963974828
C	9.1871587511	8.7900037090	1.8262637839
C	10.1347190578	9.7288598680	1.4064070495
C	11.4973350371	9.4161903523	1.4677281878
C	18.0738351507	8.9832085242	3.3608772358
C	19.0622309598	9.2746559771	2.4097390951
C	20.2861297621	9.8104575401	2.8223780228
C	20.5390997823	10.0425307845	4.1776226753
C	19.5552060082	9.7379803457	5.1259197344
C	18.3254058161	9.2184944274	4.7203715546
C	21.0049494320	1.0471430549	8.3982770708
C	20.5720467893	1.7641109359	7.2729097961
C	20.9126648042	1.3191806622	5.9855939419
C	21.6697607000	0.1566744176	5.8310091481

C	22.1031535911	-0.5618902543	6.9500658096
C	21.7742472018	-0.1081792363	8.2310821954
C	15.1305447965	-3.9499217828	8.1821481076
C	16.3903097013	-4.5465841362	8.0284663964
C	16.6127906378	-5.4499561026	6.9849076563
C	15.5868306461	-5.7620652338	6.0879322308
C	14.3280942386	-5.1727524071	6.2476569711
C	14.0972410980	-4.2749930935	7.2901032456
C	9.9258499931	-2.7755894797	11.5726893492
C	8.8823170710	-2.1593828754	12.2781755073
C	8.4316542959	-2.7124771073	13.4790811495
C	9.0152909580	-3.8783371369	13.9846189571
C	10.0544317512	-4.4927808722	13.2773593300
C	10.5090815063	-3.9493978090	12.0736122548
C	10.1568902567	-1.5870195586	5.4310232798
C	9.5044982384	-1.2765695566	4.2288487243
C	9.3758702760	-2.2451861618	3.2293041933
C	9.8898804324	-3.5312116437	3.4249621801
C	10.5402852630	-3.8409322374	4.6246325462
C	10.6763926923	-2.8772267604	5.6260000121
C	6.4162324176	4.7316590344	4.6262005953
C	7.0693396000	3.5747695309	5.0807397632
C	6.4410055419	2.3274515865	4.9625358988
C	5.1748065780	2.2374813534	4.3780587558
C	4.5245802067	3.3875905213	3.9182724608
C	5.1488641481	4.6336813201	4.0494615165
C	9.9049879765	9.0132080441	8.4309895224
C	9.1959374207	9.4485365411	9.5533386750
C	8.0308056440	8.7886373471	9.9542351793
C	7.5712664264	7.6931444135	9.2158602799
C	8.2697497707	7.2517917634	8.0889969218
C	9.4459230848	7.9093037253	7.6946813046
C	13.4488113902	12.5256687739	3.2693337808
C	12.6119895243	11.7304853563	4.0680500624
C	11.2236231296	11.9132289931	4.0213326192
C	10.6785910370	12.8892389411	3.1829546831
C	11.5092177515	13.6877307937	2.3908764546
C	12.8954530611	13.4996179078	2.4357853349
C	7.2505716399	1.4887788400	9.0306348173
C	8.0782667896	2.5787232912	9.3427249499
C	7.5868744736	3.8868757758	9.2037403135
C	6.2819186196	4.0926006954	8.7516475745
C	5.4537869098	3.0086761669	8.4422967738
C	5.9430830353	1.7071804614	8.5877146585
C	11.1589890629	8.2194819185	12.1538146542
C	10.5640883569	6.9743450562	11.9060820482
C	9.5311221432	6.5158074149	12.7388074638
C	9.0963489526	7.3060760907	13.8045751438
C	9.6836742770	8.5532984781	14.0493552549
C	10.7189074316	9.0037771869	13.2235402766
C	15.3654402266	9.1136882369	8.9000052102
C	16.3227236881	9.8202365131	8.1530163091
C	17.4035916098	10.4214216565	8.8017029264
C	17.5378445802	10.3257298840	10.1915615011
C	16.5821188276	9.6234276259	10.9329933022
C	15.4955120283	9.0217078057	10.2917280860
C	19.3482207033	7.5173041874	9.5505107529
C	20.5160776833	7.8587466355	10.2318288228
C	21.7597024992	7.7631681261	9.5983167502
C	21.8312950312	7.3178998077	8.2749472062

C	20.6665693005	6.9892391217	7.5767369462
C	19.4218455587	7.0957620690	8.2139761576
C	16.7220203489	0.4464166331	11.7773216852
C	16.2313275251	1.1006346375	12.9179037716
C	15.7157570427	0.3526923106	13.9775230763
C	15.6928027662	-1.0445924687	13.9142552858
C	16.1969962392	-1.6948215742	12.7836419908
C	16.7160526605	-0.9535851852	11.7191179911
C	12.4155147089	1.2932740833	12.8380118474
C	11.6397920749	1.9010194676	13.8374474095
C	10.9745114528	1.1068547931	14.7744260665
C	11.0686121250	-0.2878733268	14.7179577079
C	11.8388620008	-0.8901556923	13.7171180615
C	12.5173369985	-0.1045966343	12.7818536532
C	14.4319583759	5.5300621130	13.8966900125
C	13.2237902443	6.0840601464	14.3542650257
C	12.5657387522	5.5136965875	15.4501549076
C	13.0891857392	4.3761466134	16.0687619959
C	14.2974367572	3.8321599557	15.6243752215
C	14.9689107067	4.4064634990	14.5434282764
C	21.9334793172	5.0576251279	11.8452427675
C	20.6495702873	5.2745641304	12.3678943146
C	20.4591351201	6.2306878108	13.3762764043
C	21.5463766450	6.9692056266	13.8494727350
C	22.8270154009	6.7571702422	13.3286304768
C	23.0150774473	5.7975611821	12.3276529093
H	12.3851634497	-0.7181491753	-2.8213859667
H	14.6434516200	2.3965839859	-0.9013139081
H	15.0025476232	3.5006154791	-3.0960066512
H	14.0817447606	2.4683467809	-5.1830359264
H	12.7777503479	0.3587160167	-5.0405678593
H	19.3152779205	0.3359450560	3.7268019068
H	21.4045398448	1.2242469179	2.7223804791
H	21.3638027210	2.2730214627	0.4627984704
H	19.2165789568	2.3873426583	-0.8033260226
H	17.1229141343	1.5062787374	0.2067038458
H	10.6810858997	3.4088144419	0.0553410622
H	9.7527854560	5.4929288900	-0.9339668944
H	7.4475022051	6.2301468631	-0.3604286856
H	6.0849935975	4.8750299150	1.2419347303
H	7.0269608998	2.8277168694	2.2547137502
H	16.1570805983	6.1679820639	0.9902897093
H	15.4513531187	8.1554633228	-0.2938845637
H	13.3025912985	8.1221372891	-1.5781777173
H	11.8789572601	6.0786819451	-1.5291237983
H	12.5771946700	4.1008196604	-0.2000523487
H	21.1372002152	5.9877389859	4.1106718405
H	18.5815771015	5.0361255282	0.7774008659
H	20.0435185427	6.3718104668	-0.7245786497
H	22.0785433767	7.4951782681	0.1821080676
H	22.6101811210	7.3043572880	2.6126764061
H	12.0640620800	-1.8322928464	2.7039489228
H	15.9553325639	-3.3169367047	3.8115567101
H	16.0785921397	-4.5780063173	1.6706510845
H	14.1839109818	-4.4906880307	0.0541673497
H	12.1672617724	-3.1098987591	0.5915186515
H	18.8079641468	-2.8383271423	4.7624484095
H	19.1077617105	-1.9561972849	8.9649366164
H	20.4794987741	-4.0075124284	9.2989830393
H	21.0303934613	-5.4726656728	7.3559818266

H	20.1917004134	-4.8745998712	5.0849907624
H	12.9713685886	7.9171580404	1.9725269408
H	8.8605532139	6.7956715962	2.6120358718
H	8.1257360868	9.0266668325	1.7875846745
H	9.8168588322	10.7037381781	1.0439631028
H	12.2422950768	10.1488484668	1.1630418514
H	18.8793288688	9.0734610292	1.3568000137
H	21.0463690678	10.0338571054	2.0773224010
H	21.4948729389	10.4537248629	4.4944314440
H	19.7414215047	9.8928191442	6.1874276660
H	17.5632707727	8.9762812414	5.4555756132
H	20.7300782093	1.3988397575	9.3915256850
H	20.5726229157	1.8807720250	5.1169935990
H	21.9188322963	-0.1942206213	4.8312119917
H	22.6815366811	-1.4739276515	6.8216167416
H	22.1049772578	-0.6615903054	9.1079846249
H	17.2002864081	-4.2926145029	8.7073285633
H	17.5981786462	-5.8959062688	6.8706817817
H	15.7663745169	-6.4478585649	5.2633406933
H	13.5238239268	-5.3953726043	5.5489625294
H	13.1246396695	-3.8029535324	7.4062257194
H	8.4445030566	-1.2385719684	11.8958530319
H	7.6277960376	-2.2207977920	14.0232097110
H	8.6679865991	-4.3039166240	14.9230323835
H	10.5188046281	-5.3982918328	13.6625264081
H	11.3259628936	-4.4185961247	11.5274268228
H	9.0916655577	-0.2805022239	4.0841256543
H	8.8776067012	-1.9816097645	2.2978678528
H	9.7975526364	-4.2830335149	2.6445077493
H	10.9492631112	-4.8361795500	4.7844562643
H	11.1719486382	-3.1214479873	6.5636342138
H	6.9106757126	5.6980740535	4.7113253292
H	6.9518877152	1.4369125320	5.3275985911
H	4.6991733853	1.2635788901	4.2801305062
H	3.5408798575	3.3143271253	3.4602198075
H	4.6525142621	5.5348768937	3.6948684344
H	10.8178536272	9.5194675965	8.1218669833
H	9.5684436391	10.2982894012	10.1216017844
H	7.4924510298	9.1198192841	10.8393049853
H	6.6637999910	7.1752255362	9.5202023613
H	7.9169653745	6.3913723655	7.5225272964
H	14.5260583000	12.3719733747	3.3000885753
H	10.5793282964	11.2772152528	4.6262944284
H	9.5988663420	13.0205113948	3.1515425042
H	11.0836638099	14.4520663608	1.7448248306
H	13.5491216877	14.1222422974	1.8275719719
H	7.6402555492	0.4768483752	9.1301869939
H	8.2326512420	4.7346317880	9.4283551630
H	5.9115568065	5.1083128713	8.6283416189
H	4.4424224741	3.1793557862	8.0805776060
H	5.3132960032	0.8522289113	8.3486491076
H	11.9566334355	8.5649527793	11.4962879656
H	9.0691280691	5.5499899871	12.5407079442
H	8.2905637255	6.9525726073	14.4442291132
H	9.3333840057	9.1671693565	14.8755926554
H	11.1853930063	9.9701566268	13.4068292953
H	16.2106341470	9.9018838275	7.0727438047
H	18.1453895071	10.9587330348	8.2140525169
H	18.3887382147	10.7844438873	10.6904469388
H	16.6756814226	9.5312184928	12.0134630304

H	14.7480984690	8.4804575812	10.8667854606
H	18.3813701694	7.5809385168	10.0460691206
H	20.4527739938	8.1811731560	11.2682702098
H	22.6671082317	8.0147477154	10.1417302292
H	22.7956172990	7.2248484269	7.7797616324
H	20.7095699543	6.6507539586	6.5429466716
H	16.2400575437	2.1874043519	12.9579653141
H	15.3066982389	0.8685127053	14.8434803234
H	15.2735815770	-1.6187078344	14.7373621577
H	16.1801986879	-2.7806252891	12.7156556742
H	17.1034250456	-1.4552156542	10.8357748027
H	11.5765975894	2.9867170846	13.8886797738
H	10.3879550042	1.5839624843	15.5584567868
H	10.5421679306	-0.8997721295	15.4478678481
H	11.9055434398	-1.9746739819	13.6544560205
H	13.1309857820	-0.5701103749	12.0113971267
H	12.8017062652	6.9548477997	13.8574884436
H	11.6332883287	5.9516430477	15.8012648243
H	12.5747668222	3.9297023112	16.9181878316
H	14.7144150527	2.9460783925	16.0963754466
H	15.9170534271	4.0022422236	14.1942256134
H	22.0726564909	4.3264556593	11.0505840010
H	19.4586998253	6.4007815137	13.7718462203
H	21.3883697686	7.7135091292	14.6274399366
H	23.6682746679	7.3381076519	13.6983596954
H	24.0069574525	5.6272686172	11.9130911342

Table S3. Coordinates of $Au_{44}(SC_6H_5)_{28}$ employed in the calculations.

Au	16.6349190000	15.0443270000	9.8810050000
Au	16.6349190000	17.9942810000	9.8810050000
Au	19.0013350000	16.5442330000	8.9296620000
Au	18.3246910000	16.6347700000	11.7813200000
Au	16.1351080000	17.9494300000	12.7721870000
Au	13.4615640000	16.5730210000	13.8915080000
Au	11.4049970000	15.1323610000	11.6548430000
Au	12.5374090000	15.1619830000	8.6921230000
Au	15.7895610000	15.0578870000	12.6306910000
Au	17.6040380000	16.4639180000	14.9527270000
Au	20.2113910000	18.4265210000	13.8432880000
Au	20.6096790000	18.0564470000	10.6726710000
Au	18.1691910000	19.4378630000	11.7840870000
Au	20.7533830000	21.8105930000	11.2848980000
Au	22.7054200000	20.6682440000	8.5000360000
Au	21.1902890000	17.9602780000	8.0024280000
Au	19.0942780000	13.8101910000	8.9399380000
Au	21.1721350000	11.4787650000	10.7442100000
Au	21.6834240000	12.6307100000	13.9515840000
Au	19.8828300000	15.3958350000	13.6551540000
Au	18.4495310000	13.8719390000	11.8338870000
Au	16.6349190000	11.7274280000	9.8810050000
Au	18.9398990000	19.3195810000	8.8640520000
Au	16.6349190000	21.5944730000	9.8810050000
Au	14.2685030000	16.5442330000	10.8323480000
Au	14.9451470000	16.6347700000	7.9806900000
Au	17.1347300000	17.9494300000	6.9898230000
Au	19.8082750000	16.5730210000	5.8705030000
Au	21.8648410000	15.1323610000	8.1071670000
Au	20.7324290000	15.1619830000	11.0698880000
Au	17.4802780000	15.0578870000	7.1313190000
Au	15.6658010000	16.4639180000	4.8092830000
Au	13.0584470000	18.4265210000	5.9187220000
Au	12.6601590000	18.0564470000	9.0893390000
Au	15.1006470000	19.4378630000	7.9779240000
Au	12.5164550000	21.8105930000	8.4771120000
Au	10.5644190000	20.6682440000	11.2619740000
Au	12.0795490000	17.9602780000	11.7595820000
Au	14.1755610000	13.8101910000	10.8220720000
Au	12.0977040000	11.4787650000	9.0178010000
Au	11.5864140000	12.6307100000	5.8104260000
Au	13.3870080000	15.3958350000	6.1068560000
Au	14.8203080000	13.8719390000	7.9281230000
Au	14.3299400000	19.3195810000	10.8979580000
S	14.4314100000	18.6330450000	14.3076950000
S	12.1356460000	14.6506800000	13.7899310000
S	10.4082090000	15.5727370000	9.6161940000
S	16.8072630000	14.2814410000	14.6772450000
S	18.5247560000	18.5245680000	15.4258300000
S	21.9650140000	18.9772520000	12.4144950000
S	18.7909020000	21.6516320000	12.4342570000
S	22.7355800000	22.2294820000	10.1813880000
S	22.8392850000	19.1587420000	6.7665120000
S	19.9761960000	11.6112330000	8.7782850000
S	22.3616360000	10.8998730000	12.5923530000
S	21.2872430000	14.4650170000	15.3076530000
S	18.0325770000	11.5257030000	11.6951580000
S	18.7014540000	21.6808370000	8.8494280000

S	18.8384280000	18.6330450000	5.4543150000
S	21.1341930000	14.6506800000	5.9720800000
S	22.8616290000	15.5727370000	10.1458160000
S	16.4625750000	14.2814410000	5.0847650000
S	14.7450820000	18.5245680000	4.3361800000
S	11.3048240000	18.9772520000	7.3475150000
S	14.4789370000	21.6516320000	7.3277530000
S	10.5342580000	22.2294820000	9.5806230000
S	10.4305540000	19.1587420000	12.9954980000
S	13.2936420000	11.6112330000	10.9837250000
S	10.9082020000	10.8998730000	7.1696570000
S	11.9825950000	14.4650170000	4.4543570000
S	15.2372620000	11.5257030000	8.0668530000
S	14.5683850000	21.6808370000	10.9125820000
C	14.8238820417	18.6936750285	16.0477670277
C	14.5436125903	17.6398563095	16.9310981035
C	14.6535958553	17.8353110782	18.3101381586
C	15.0503073293	19.0751654532	18.8234933657
C	15.3671557944	20.1134910189	17.9403447281
C	15.2614867940	19.9277211483	16.5599412206
C	10.8300557671	15.0706891880	14.9454765729
C	11.0930599130	14.8423961089	16.3064454124
C	10.0992682232	15.0898836830	17.2556975635
C	8.8450859514	15.5662273641	16.8579122896
C	8.5885453110	15.7921567088	15.5021894134
C	9.5753657344	15.5438078668	14.5442399915
C	9.1039499611	14.3686929994	9.3949603007
C	8.0162379503	14.3806355202	10.2879739550
C	6.8718288246	13.6276622057	10.0027673709
C	6.8213349896	12.8342930444	8.8476504355
C	7.9176988377	12.8037750786	7.9791006462
C	9.0488004912	13.5813834738	8.2421963461
C	15.6219302864	13.9225105510	15.9569848607
C	15.7454462475	14.4850231049	17.2366118589
C	14.9276147053	14.0379098348	18.2758724252
C	13.9896652643	13.0240638698	18.0517389868
C	13.8633404881	12.4721882981	16.7729248107
C	14.6675246877	12.9201227406	15.7231450616
C	19.2523413760	18.3569579739	17.0559694615
C	20.6229456748	18.5235053227	17.3017553354
C	21.1026531690	18.4852027705	18.6157251869
C	20.2285113323	18.2893698587	19.6920379468
C	18.8623318606	18.1179996837	19.4381190263
C	18.3741730413	18.1466505837	18.1303431441
C	23.4287305188	18.0370935041	12.8192286143
C	24.5827890028	18.2650251711	12.0454857953
C	25.8131265645	17.7397089041	12.4553052975
C	25.8954329143	16.9743651701	13.6251654335
C	24.7366106869	16.7060944605	14.3638142584
C	23.5052198586	17.2315431881	13.9649343244
C	19.1556593472	21.7543582753	14.1818668880
C	20.4625742016	21.7389627515	14.6949748101
C	20.6699082218	21.8338248610	16.0730983094
C	19.5840879820	21.9454490008	16.9478660078
C	18.2854946205	21.9832916857	16.4319356491
C	18.0661464712	21.8861138652	15.0556687131
C	22.4794792505	23.7911045217	9.3160174114
C	21.4799699230	24.6882235820	9.7162855745
C	21.3507446144	25.9237527905	9.0762725246
C	22.2134079792	26.2761625385	8.0349014468

C	23.2093766456	25.3788861175	7.6347841060
C	23.3480854984	24.1427732462	8.2708257772
C	24.4319534683	18.3667860498	7.0857964567
C	24.5475621773	16.9837036730	6.9039336824
C	25.7523113919	16.3320209900	7.1757012333
C	26.8533281469	17.0649432506	7.6130834299
C	26.7635922094	18.4551835579	7.7433386106
C	25.5582043653	19.1115369144	7.4816111861
C	21.0990820453	11.2644364759	7.4334427651
C	20.6835748754	11.5094552949	6.1153058130
C	21.4926388025	11.1250627521	5.0434958517
C	22.7161584094	10.4872866425	5.2682805440
C	23.1289169000	10.2358232363	6.5807127100
C	22.3280132554	10.6200089930	7.6590044988
C	21.5159715422	9.5117397076	13.3853772339
C	22.1027584262	8.9960263982	14.5529448380
C	21.5162927087	7.9092397784	15.2067092727
C	20.3441717903	7.3305168317	14.7062849067
C	19.7623527395	7.8481110900	13.5458701036
C	20.3460160590	8.9310195917	12.8803646095
C	20.2667513471	13.8849364236	16.6608033423
C	20.0820687800	12.5188384568	16.9304601401
C	19.3641543204	12.1208891990	18.0619273839
C	18.8197976629	13.0731608730	18.9288683606
C	18.9990427705	14.4324629317	18.6544322693
C	19.7172507453	14.8411310826	17.5292866338
C	17.1646909564	10.8547033053	13.0968528172
C	16.0806489928	9.9783286488	12.9299153870
C	15.5466602076	9.3189700217	14.0412235703
C	16.0693563773	9.5458472883	15.3189142632
C	17.1381857718	10.4336470602	15.4822153990
C	17.6958017297	11.0803032928	14.3782130440
C	18.6007778802	22.3496470966	7.2018958888
C	19.5642911818	21.9448731643	6.2619839555
C	19.6278200689	22.5744023504	5.0173471495
C	18.7420068669	23.6114577772	4.7039504670
C	17.7826041044	24.0111445131	5.6415066315
C	17.7034665639	23.3823093877	6.8875417948
C	18.5023955311	18.7247037580	3.7043689902
C	18.9686631321	17.7615318559	2.7963420207
C	18.8730501476	17.9872048022	1.4210828218
C	18.3170154862	19.1743981171	0.9337071183
C	17.8404350559	20.1294598050	1.8388328605
C	17.9253796344	19.9092088780	3.2153293378
C	22.5651103840	14.9266749453	4.9296989929
C	23.6237697792	14.0096802908	5.0398108980
C	24.7599303075	14.1651962828	4.2435968297
C	24.8474051834	15.2258324877	3.3342838992
C	23.7863911622	16.1281189061	3.2186136940
C	22.6441313834	15.9821322257	4.0110969928
C	23.9422043578	14.1683507138	10.4420324963
C	24.8416249620	14.2632382231	11.5154185403
C	25.8297982821	13.2869152408	11.6893626400
C	25.9020114551	12.1918962153	10.8209244352
C	24.9671812083	12.0721164790	9.7860228462
C	23.9965762118	13.0555942636	9.5890095961
C	17.5650503405	13.8673629631	3.7521568975
C	17.7531346491	14.6929468682	2.6367448607
C	18.5122182472	14.2410491369	1.5560561741
C	19.0680361984	12.9578508295	1.5652395358

C	18.8496381403	12.1217208345	2.6654926243
C	18.1067356709	12.5717156010	3.7586254494
C	14.0223811745	18.3516150142	2.7038985923
C	14.9002986169	18.1541825607	1.6276625776
C	14.4114574856	18.1460157825	0.3200451709
C	13.0457531823	18.3239410982	0.0697320620
C	12.1718428836	18.5118571899	1.1476571664
C	12.6534633766	18.5311252985	2.4607731066
C	9.9394086361	17.8800365192	7.0379313327
C	8.8281670893	17.9879920404	7.8888755160
C	7.7102977638	17.1836757730	7.6647581023
C	7.6918021399	16.2739805743	6.6029845858
C	8.7967502096	16.1755039337	5.7529960361
C	9.9176291215	16.9791853824	5.9653948480
C	14.0741985689	21.7543778212	5.5873285807
C	12.7563473992	21.7404412888	5.1011945672
C	12.5177202325	21.8561020751	3.7298510593
C	13.5824450710	21.9870790338	2.8325883183
C	14.8917868392	22.0201228823	3.3208192313
C	15.1415549668	21.9010032919	4.6900002415
C	10.9066615631	23.8178245517	10.3556770164
C	10.6207788095	24.9840589135	9.6288924776
C	10.8801189654	26.2374001093	10.1938260884
C	11.4248850511	26.3337326916	11.4785824434
C	11.7219651421	25.1700210364	12.1941471596
C	11.4637665448	23.9161129213	11.6363770531
C	8.7286617887	18.5927266814	12.8479392171
C	8.2049749515	17.9353926652	11.7264314234
C	6.8380651105	17.6354038106	11.6633021606
C	5.9678554558	17.9777826396	12.7114277049
C	6.5161338926	18.5921454630	13.8476660758
C	7.8792606945	18.8959741349	13.9226472634
C	11.9932539681	11.5702380054	12.2210957990
C	12.3093683905	11.0683082859	13.4913062499
C	11.3195311692	10.9924027839	14.4765174719
C	10.0182785002	11.4308890263	14.2142627593
C	9.7022133958	11.9249480523	12.9449781196
C	10.6796429253	11.9902725609	11.9500056132
C	11.7499846727	9.4698574925	6.4660164066
C	11.7160252100	9.2809513909	5.0751155808
C	12.3075925480	8.1487187616	4.5120904457
C	12.9219878617	7.1944508549	5.3276318730
C	12.9404847698	7.3743213463	6.7135887523
C	12.3618880255	8.5115768204	7.2860785782
C	13.0577319851	14.0456197799	3.0869153060
C	12.9752961231	14.8710428844	1.9551803419
C	13.7884981464	14.6126730051	0.8485772770
C	14.6888149571	13.5432030746	0.8656471967
C	14.7763810532	12.7296878037	2.0006923390
C	13.9648352157	12.9773144589	3.1099445933
C	16.0324110586	10.7314249750	6.6861421566
C	17.3307711732	10.2054439311	6.7435351353
C	17.8239501386	9.4558230068	5.6700293404
C	17.0342607575	9.2305278105	4.5380330795
C	15.7369997109	9.7542121322	4.4847390467
C	15.2334784457	10.4963167319	5.5536599143
C	14.6752630496	22.3628875537	12.5536750287
C	13.7455380529	21.9347526697	13.5166611061
C	13.6461666077	22.6062614588	14.7375432451
C	14.4741022180	23.7011639586	15.0111894489

C	15.4147660652	24.1117654485	14.0592078049
C	15.5216826452	23.4479294277	12.8339752911
H	14.2201140099	16.6788432939	16.5337545711
H	14.4189598880	17.0111735700	18.9806774012
H	15.6922193313	21.0771302655	18.3274875020
H	15.4834426698	20.7428195007	15.8760929905
H	15.1237690038	19.2281295720	19.8989124953
H	12.0677112564	14.4690677491	16.6138323474
H	10.3086203681	14.9100801297	18.3081186702
H	7.6249711770	16.1777683968	15.1788828864
H	9.3810296997	15.7305486874	13.4893063518
H	8.0740304626	15.7627864596	17.5993749356
H	8.0656828424	15.0005727195	11.1809791640
H	6.0083379711	13.6673965352	10.6694179400
H	7.8977535128	12.1893650448	7.0816359735
H	9.8816813601	13.6139526563	7.5395570242
H	5.9222497793	12.2586838809	8.6272049526
H	16.4890365351	15.2608743458	17.4078145010
H	15.0338151720	14.4773239177	19.2656368006
H	13.1279867578	11.6940625388	16.5838618249
H	14.5676007238	12.4938747625	14.7275212755
H	13.3605790198	12.6700310450	18.8654904315
H	21.3028334734	18.6800470808	16.4649582164
H	22.1685135770	18.6085420602	18.7959471604
H	18.1667183119	17.9618295227	20.2597060963
H	17.3133759326	18.0048546412	17.9387017522
H	20.6045519806	18.2671395444	20.7138603110
H	24.5131509861	18.8588491314	11.1348156670
H	26.7023981573	17.9244761231	11.8552164073
H	24.7860349163	16.0883391151	15.2576916435
H	22.6043893294	17.0311478862	14.5431613498
H	26.8490186394	16.5688767808	13.9501273990
H	21.3041830539	21.6420592823	14.0105883173
H	21.6850675368	21.8050350227	16.4625952453
H	17.4350449565	22.0680945523	17.1043744184
H	17.0557286796	21.8994177192	14.6536282801
H	19.7484962085	21.9831987462	18.0217558165
H	20.8001033440	24.4092688663	10.5200666497
H	20.5671497153	26.6072906390	9.3945222082
H	23.8830856438	25.6391964315	6.8215833644
H	24.1172835776	23.4425655662	7.9517666198
H	22.1089900628	27.2407660595	7.5411985713
H	23.6842998023	16.4140531506	6.5761281218
H	25.8138370962	15.2529549506	7.0578510771
H	27.6441741418	19.0236243955	8.0462052465
H	25.4780511650	20.1902215162	7.5977950329
H	27.7933829731	16.5625067696	7.8366011547
H	19.7386895143	12.0136666744	5.9324146590
H	21.1696561716	11.3524093870	4.0300944438
H	24.0773107506	9.7368365580	6.7706171150
H	22.6421193969	10.4166007515	8.6817726865
H	23.3462076909	10.2005883354	4.4297472852
H	23.0095063761	9.4513969170	14.9454938782
H	21.9786863673	7.5134957442	16.1083870899
H	18.8448431488	7.4149451348	13.1530084632
H	19.8894185202	9.3370435950	11.9801738777
H	19.8948319947	6.4765251017	15.2114602705
H	20.5103353462	11.7740435729	16.2597441367
H	19.2414438089	11.0590694620	18.2669444341
H	18.5851610343	15.1899430794	19.3164546752

H	19.8554500799	15.8997781892	17.3279439184
H	18.2684648341	12.7611222443	19.8136469918
H	15.6656420060	9.8188567062	11.9363435735
H	14.7161314001	8.6295093122	13.9032493028
H	17.5394220515	10.6362009894	16.4725275440
H	18.5374534368	11.7594828680	14.5031718401
H	15.6429128970	9.0402507233	16.1823440031
H	20.2557727715	21.1429116557	6.5115161209
H	20.3708950877	22.2481807273	4.2931547969
H	17.0818479004	24.8067209474	5.3999208364
H	16.9481758598	23.6781396139	7.6123362573
H	18.7962195459	24.1026323419	3.7344770665
H	19.4118119848	16.8416069237	3.1749064864
H	19.2462594560	17.2316445550	0.7325042886
H	17.3989133596	21.0534553707	1.4717229522
H	17.5704564242	20.6575066245	3.9179594172
H	18.2516045970	19.3513060355	-0.1383229097
H	23.5572561018	13.1935163124	5.7568257146
H	25.5873502871	13.4650439736	4.3452442659
H	23.8488089162	16.9605675912	2.5210809009
H	21.8276069196	16.7003159874	3.9457720220
H	25.7452698536	15.3529837575	2.7326272866
H	24.7848435923	15.1119227482	12.1910506698
H	26.5504910791	13.3889997200	12.4997588171
H	24.9991211371	11.2190511012	9.1114586407
H	23.2899172381	12.9733926096	8.7635287932
H	26.6816209543	11.4429231167	10.9482485402
H	17.2833469652	15.6737798933	2.6161502233
H	18.6516028708	14.8911904669	0.6946677023
H	19.2430454938	11.1067224958	2.6722868254
H	17.9098279021	11.9082879834	4.5973850365
H	19.6518584556	12.6078640205	0.7175802114
H	15.9627306657	18.0182478301	1.8150960007
H	15.1048105501	17.9981635562	-0.5044779640
H	11.1071925947	18.6464259840	0.9687973942
H	11.9769243787	18.6912057134	3.2992663096
H	12.6695593791	18.3140845030	-0.9521166612
H	8.8514072336	18.6881989385	8.7225513594
H	6.8514586306	17.2595340887	8.3279182553
H	8.7968706697	15.4678598276	4.9270499696
H	10.7808308100	16.9028468277	5.3076976331
H	6.8227365265	15.6393890719	6.4469151489
H	11.9294826782	21.6340075337	5.8019132635
H	11.4934148949	21.8332623153	3.3636674691
H	15.7275664494	22.1260328639	2.6329075838
H	16.1592672858	21.9203052252	5.0719117769
H	13.3938008274	22.0474752045	1.7636911646
H	10.2074702122	24.9067212829	8.6257728490
H	10.6568556253	27.1429678899	9.6312151142
H	12.1651896812	25.2256168023	13.1865976388
H	11.7045749369	23.0122770784	12.1896141986
H	11.6174771942	27.3107332323	11.9171031011
H	8.8683002536	17.6670156473	10.9061385264
H	6.4672894666	17.1312133608	10.7731662255
H	5.8661058289	18.8532460876	14.6821170103
H	8.2855257215	19.3830291940	14.8061214073
H	4.8985591679	17.7689675657	12.6500409126
H	13.3237221673	10.7362965313	13.7022436911
H	11.5672999131	10.5848288494	15.4543658504
H	8.6946460449	12.2674188477	12.7186826908

H	10.4236514875	12.3603714185	10.9579648011
H	9.2585872435	11.3882929277	14.9909927182
H	11.2443031040	10.0288550718	4.4406595699
H	12.2863082324	8.0152404852	3.4325592204
H	13.4055294537	6.6229680306	7.3509419329
H	12.3931427365	8.6667279409	8.3630585948
H	13.3800025181	6.3107349433	4.8883428267
H	12.2927442293	15.7174918505	1.9477972695
H	13.7171503728	15.2587288524	-0.0239522856
H	15.4942494793	11.9124349760	2.0362685908
H	14.0486222573	12.3645272752	4.0023477464
H	15.3232520441	13.3502311019	0.0029795166
H	17.9443410063	10.3756964735	7.6263764655
H	18.8359635637	9.0588242129	5.7226599759
H	15.1038974444	9.5727363389	3.6185227786
H	14.2081136125	10.8635562089	5.5365947285
H	17.4237528839	8.6495877370	3.7051435678
H	13.1007287686	21.0847655083	13.3007566033
H	12.9212706791	22.2655397078	15.4740234324
H	16.0738837207	24.9507120618	14.2712587474
H	16.2535893003	23.7625882931	12.0924866106
H	14.3909345174	24.2254748025	15.9605875260

Table S4. Coordinates of $Au_{52}(SC_6H_5)_{32}$ employed in the calculations.

Au	8.2561182900	5.6467683500	28.9800242500
Au	6.3969108600	3.5156198700	29.4298932800
Au	9.0366662800	2.8027922800	28.4071141300
Au	7.1627021200	4.1616684400	26.6768486500
Au	7.5301127500	5.0271351200	31.7330688800
Au	8.2721399600	2.1543717700	30.9986673100
Au	5.3475992300	2.0334696100	26.9421560200
Au	9.9182576800	3.4293604700	25.7194350800
Au	5.5941203100	6.3689247600	29.9528179600
Au	7.0514783600	4.3175062300	34.3323121300
Au	10.8253738200	4.1836451100	23.2701481700
Au	3.5320096800	7.8333558300	28.0226106900
Au	12.9329496100	2.6293905700	24.4159684200
Au	6.7051294500	3.4640241800	20.7785658800
Au	8.7203743700	6.6188577100	34.2515664100
Au	4.7099458800	8.9393121100	30.3757717400
Au	12.1795714400	5.7832334100	25.2964813000
Au	2.7181769200	6.8083444800	31.2447495100
Au	9.9704736500	4.5355028000	20.3133167200
Au	7.8918095200	1.3718374100	26.1077835600
Au	13.0388076200	-0.9337357800	26.6076380300
Au	6.1922765000	2.7354793600	24.4236584900
Au	2.3771029000	3.4774843600	33.8132324900
Au	4.4823009600	4.9059120200	27.8688093100
Au	10.4278538500	7.8980360300	28.5724506100
Au	7.0329868900	0.8193643200	28.9800242500
Au	10.6034015000	-3.3130596800	25.6617595700
Au	10.0327961500	4.4431676500	30.7910354500
Au	4.7784761000	3.6776356200	36.2855896100
Au	5.1294337800	5.6318806100	24.7120360700
Au	6.4827107100	6.8355158900	27.0998024300
Au	5.8030805000	2.9582847200	32.3136690700
Au	10.9973908300	4.7773509300	27.8726543500
Au	9.0033866500	6.2045732000	26.3423306600
Au	7.3303416400	8.5479455700	29.5721595500
Au	3.8974334000	4.2526294400	30.4718976000
Au	5.7106635500	-0.8861945800	26.6114830600
Au	9.5925811900	-0.0335646500	27.7534582800
Au	8.9734922600	2.1494069000	23.4239495500
Au	9.3050227000	7.0664498800	31.1755388900
Au	8.2572319700	4.7522576300	23.9161139500
Au	11.5828503800	2.1439492400	27.4843058700
Au	10.5158670500	0.4602105600	25.2311157200
Au	5.0572737200	5.5539116300	32.9134944400
Au	4.2571326300	1.2561772500	29.9874232600
Au	8.3619214800	-1.2490188800	25.5887039100
Au	6.6438944600	-0.0508885200	23.3624290000
Au	9.8021138300	3.4244830100	33.6517410500
Au	11.3794649700	1.1817071900	30.2911809800
Au	6.5607476400	7.9119741800	32.0906570800
Au	5.8064391800	11.7069597000	30.7833453900
Au	8.9746581900	10.5589381300	31.4754515800
S	12.1343922000	6.5156855100	29.0684600400
S	10.6574536900	5.4185408700	34.3592273700
S	7.0394549400	3.7778572200	36.6431778100
S	13.9951930500	4.3901230800	25.3810720600
S	10.6890329200	7.5624967100	25.4464376400
S	11.9099022100	5.0786714700	21.4322217300

S	12.8257873800	-3.1532329000	26.1808392100
S	8.4342650200	-3.5754698100	24.8812175900
S	12.0438085300	0.8272611800	23.4585548600
S	2.4878933400	3.5907602600	36.1087180300
S	3.6404232400	11.0323531300	30.8640911100
S	3.2501999300	1.8507721300	27.9918504100
S	4.7472750900	-0.4909649400	24.5736148300
S	11.0073579500	-0.9442027200	29.4491184500
S	12.0930959400	3.3672340100	30.7602751800
S	8.2298205000	0.6139007100	21.7782748300
S	9.3612004300	1.3036700200	32.9211845100
S	1.7586665500	6.9403718100	29.1645859000
S	8.1468866000	3.9891073500	19.0175401300
S	3.3458896600	4.6601742600	25.8040258400
S	5.1447912700	0.6698316900	32.0522067400
S	6.6222630200	-1.5301366200	28.6301261200
S	6.9148518400	8.0089221100	34.4092128200
S	5.1099595000	3.2526662600	22.3819452300
S	6.9178595800	6.6863807100	23.6046661700
S	5.2538249800	8.8817116100	26.9037056800
S	3.0061208700	6.6446867000	33.5479251200
S	13.7090498300	1.1066773100	27.2382236700
S	10.1612068800	8.8539711500	32.4444002400
S	8.0007175000	12.3419009800	30.4642075300
S	2.0934847000	3.2298341700	31.5754224700
S	8.8491325100	9.2916175900	27.9418649700
C	13.6356553770	7.1436787385	28.3451877317
C	13.7152307707	8.3750760898	27.6772570902
C	14.8108646368	6.4247820047	28.6251874487
C	14.9635971482	8.9005738787	27.3348497926
C	16.0534315668	6.9536627738	28.2730151064
C	16.1359319471	8.2012810185	27.6418142973
C	11.2102346071	5.2947386753	36.0450259470
C	11.6813190618	6.4691207893	36.6500115670
C	11.2694635582	4.0740663095	36.7218572834
C	12.1604076704	6.4204211294	37.9604331898
C	11.7446820798	4.0426307702	38.0353055887
C	12.1733825932	5.2133120572	38.6759256385
C	7.2037605099	5.0767299896	37.8782701198
C	8.3062176551	5.9414473473	37.8647990086
C	6.3307095108	5.0857904995	38.9848868005
C	8.5507804109	6.7779147524	38.9622008164
C	6.5692835857	5.9419271133	40.0595833312
C	7.6875512729	6.7831783016	40.0594699431
C	15.0160745794	5.1977152706	24.1444258220
C	15.1978517829	4.6804480626	22.8556235245
C	15.6286812866	6.4062828853	24.5137133950
C	15.9708840377	5.3896136665	21.9314710490
C	16.3999810450	7.1034835922	23.5836763947
C	16.5666193160	6.6026290356	22.2873787191
C	10.2327385561	8.6444440083	24.1099778134
C	10.2907782328	10.0073798059	24.4563592962
C	9.8152786365	8.2604658915	22.8321101919
C	9.9392755582	10.9820403477	23.5213707205
C	9.4894772008	9.2469871612	21.8983908273
C	9.5460618182	10.6029283731	22.2348265976
C	12.2088476569	6.8340438235	21.2915666387
C	11.8895767775	7.5317767561	20.1141997229
C	12.9164696485	7.4850500823	22.3103578384
C	12.2379896523	8.8791907024	19.9943964833

C	13.2523923365	8.8329895456	22.1844988026
C	12.9072032177	9.5399052694	21.0302528386
C	13.8293735017	-3.6531397588	24.7912424876
C	15.2217027056	-3.6684579429	24.9768479093
C	13.2800060228	-4.1865512609	23.6190263153
C	16.0456010273	-4.2192037223	23.9931593071
C	14.1134206923	-4.7185971259	22.6314681825
C	15.4984600848	-4.7392356070	22.8149647825
C	8.4395588386	-3.8757637651	23.1214295131
C	7.4212739533	-3.3575264504	22.3089492963
C	9.3380730672	-4.8090759785	22.5813598120
C	7.3432773673	-3.7169611822	20.9608118174
C	9.2446154272	-5.1748170091	21.2367481643
C	8.2498016371	-4.6345814343	20.4154342692
C	13.5304845718	-0.0735256335	22.9727601708
C	14.6794136464	-0.2026904524	23.7664005242
C	13.5571875208	-0.4967240009	21.6356281944
C	15.8340892397	-0.7742699045	23.2310848147
C	14.7190048945	-1.0681736636	21.1079589567
C	15.8592834992	-1.2106653669	21.9028693452
C	1.7931069544	2.0440988256	36.6780429763
C	0.5392960020	1.6605456477	36.1764033615
C	2.3918901950	1.2949878161	37.6990204954
C	-0.0939912525	0.5186606873	36.6639487877
C	1.7461898393	0.1540449959	38.1845738873
C	0.5013639007	-0.2344353300	37.6833911599
C	2.6784496596	11.3747712486	32.3106098127
C	1.5458277248	10.5762778976	32.5456986325
C	2.9239247853	12.5061427425	33.1022592925
C	0.6750696431	10.9054808710	33.5840549867
C	2.0402855130	12.8261887359	34.1364452733
C	0.9184220953	12.0278871917	34.3837984709
C	2.4804123664	0.3824539489	27.3118332421
C	2.6071983690	-0.8898401141	27.8892845548
C	1.7401164271	0.5398186310	26.1293480159
C	2.0052252870	-1.9952907071	27.2824351496
C	1.1430163650	-0.5716518333	25.5301092779
C	1.2768062630	-1.8417378434	26.0994459866
C	4.2795000961	-2.1827519385	24.0245283664
C	3.3292595615	-2.2723927706	22.9957423408
C	4.7899152389	-3.3720453225	24.5825344068
C	2.8901946633	-3.5260341664	22.5507242980
C	4.3527656088	-4.6225564453	24.1264615237
C	3.3928958017	-4.7064639668	23.1124306850
C	10.1657109726	-1.8802441819	30.7082196393
C	10.5885627312	-1.7954476879	32.0460349833
C	9.1840322366	-2.8091880860	30.3409993334
C	10.0236283323	-2.6348917994	33.0063220465
C	8.6143885111	-3.6369495382	31.3127408857
C	9.0359859525	-3.5580990824	32.6434257721
C	12.9517464397	3.3875431373	32.3202785851
C	12.9655394264	2.3132015800	33.2212930046
C	13.6982676174	4.5417505596	32.6004642250
C	13.7154926506	2.4040078604	34.3967226916
C	14.4355068492	4.6301296909	33.7820953413
C	14.4459210235	3.5613393243	34.6855027411
C	9.4068839852	-0.5668417648	21.1618983055
C	10.1200692784	-1.4908275847	21.9310065400
C	9.5787259348	-0.5270877988	19.7679683007
C	10.9750347978	-2.3891780298	21.2922503364

C	10.4301612474	-1.4383789392	19.1325315387
C	11.1239050396	-2.3788131490	19.9039013182
C	8.3430483942	0.4824547022	34.1412417066
C	7.6476489861	-0.6789399815	33.7712342249
C	8.3685909913	0.8746849914	35.4887847943
C	7.0021004315	-1.4403041293	34.7489983691
C	7.7067570457	0.1164635689	36.4595372801
C	7.0286926841	-1.0516371437	36.0950180502
C	0.6195685934	8.3117407428	29.4365386772
C	-0.5196180123	8.0591029575	30.2164082450
C	0.8262810357	9.5887255226	28.8976606454
C	-1.4319024425	9.0862628548	30.4661415802
C	-0.0908149638	10.6113206945	29.1554550893
C	-1.2195535357	10.3669704177	29.9425486337
C	7.5758070835	5.4207424893	18.1139902855
C	6.2412550496	5.4698077441	17.6901470637
C	8.5033357224	6.3729546914	17.6722839167
C	5.8785215654	6.3734626962	16.6734455521
C	8.1311236441	7.2796648470	16.6618460835
C	6.8120532465	7.3011431601	16.2035720003
C	1.9328254043	5.7486665973	25.7599471434
C	1.9559228243	7.0268555142	25.1842646688
C	0.7452267630	5.2705051750	26.3374678716
C	0.8016781934	7.8150858084	25.1851893798
C	-0.3984787660	6.0710090183	26.3468876422
C	-0.3766171125	7.3457726774	25.7716056414
C	3.7889052103	0.0798641565	33.0444312664
C	2.6204497961	-0.4255455530	32.4530197546
C	4.0066382617	-0.0800823267	34.4206431942
C	1.6958981211	-1.1246874814	33.2304274491
C	3.0917699847	-0.8090711587	35.1803803435
C	1.9399064468	-1.3384945389	34.5902192053
C	5.4326219577	-2.5957556304	29.4407439582
C	5.2493017468	-2.5803788108	30.8304238883
C	4.8619885894	-3.6336677250	28.6794074685
C	4.5159446412	-3.6024547391	31.4458646357
C	4.1715898527	-4.6762753315	29.3066039553
C	3.9996868629	-4.6685854795	30.6972986142
C	7.6061659687	9.5628317589	34.9762887985
C	8.3603326240	9.5656088450	36.1635425457
C	7.2881756746	10.7747753221	34.3502752670
C	8.7958977219	10.7770743917	36.7016975887
C	7.7110566713	11.9838486190	34.9123429842
C	8.4594420328	11.9922662319	36.0958407256
C	3.9171574036	2.0347500182	21.8574406915
C	2.7568805666	1.8871827046	22.6367046558
C	4.0282552199	1.3482903288	20.6360697576
C	1.7063153541	1.0953336022	22.1696488271
C	2.9658370788	0.5681754442	20.1693264163
C	1.7984984060	0.4439539263	20.9327773409
C	6.1373084324	7.1326398781	22.0715331097
C	6.9183595951	7.4036056416	20.9444256361
C	4.7725597465	7.4673030639	22.0740585695
C	6.3520715130	8.0950310109	19.8764104573
C	4.2055480880	8.1238732112	20.9858344955
C	5.0054240177	8.4694541004	19.8970601742
C	4.9283428455	9.5169323741	25.2794691421
C	6.0408023338	9.7661077880	24.4553364427
C	3.6575154494	9.9766084985	24.9034493547
C	5.8691088187	10.4568020449	23.2550324846

C	3.4998331645	10.6708258992	23.7005539099
C	4.6024095034	10.9110463763	22.8738101847
C	3.5344837032	7.9570973160	34.6243620264
C	3.9016971770	9.2301176260	34.1911163281
C	3.5920126521	7.6286148769	35.9916676674
C	4.2760779130	10.2015212544	35.1197437238
C	4.0154735036	8.5959390973	36.8988810604
C	4.3425123642	9.8871367645	36.4770318956
C	14.5134206955	1.0837131421	28.8318400271
C	15.0872004109	2.2983277095	29.2440810117
C	14.5884560942	-0.0449760367	29.6593587962
C	15.7231325883	2.3810108048	30.4830453172
C	15.2309594287	0.0492372207	30.8977043762
C	15.7939694140	1.2587338202	31.3159488267
C	11.8728204697	9.0933022579	31.9749458559
C	12.3647114386	10.3216742140	31.5061507198
C	12.7560583322	8.0155172579	32.1515749687
C	13.7170204044	10.4550269465	31.1782757476
C	14.1067205020	8.1634975892	31.8310073464
C	14.5922388470	9.3757449746	31.3297611542
C	8.3643223544	13.9445864440	31.2360609504
C	9.2317149673	14.0144216920	32.3353759266
C	7.9193580708	15.1485227871	30.6348986587
C	9.6569072627	15.2584589493	32.8150080580
C	8.3480069961	16.3926789206	31.1248573615
C	9.2267060923	16.4484465498	32.2148047526
C	0.5219748138	3.8889306471	31.0746626874
C	0.1371383778	3.6678231964	29.7414160831
C	-0.3718272831	4.4930863368	31.9715600954
C	-1.1456921985	4.0199137701	29.3231970050
C	-1.6561469924	4.8366187229	31.5415382581
C	-2.0503049646	4.5981091085	30.2205788808
C	9.4941257627	10.9468945972	27.9273602867
C	10.7199352214	11.2925187294	28.5128296951
C	8.7722453130	11.8985292609	27.1889675633
C	11.2072281773	12.5945439555	28.3861044941
C	9.2718349209	13.1945473142	27.0620502974
C	10.4841167632	13.5505062441	27.6657822269
H	12.7991811889	8.9166527346	27.4452726508
H	14.7389102648	5.4646095286	29.1337282947
H	15.0165055783	9.8629971096	26.8294837438
H	16.9579556929	6.3910758997	28.4940020307
H	17.1056137698	8.6171372918	27.3789486626
H	11.6435648719	7.4153006896	36.1140505607
H	10.9221250707	3.1650519650	36.2347460466
H	12.5122809581	7.3375031236	38.4282795213
H	11.7567580672	3.0989360023	38.5776742891
H	12.5074508920	5.1859993013	39.7142295001
H	8.9711037833	5.9466633550	37.0039051797
H	5.4759541072	4.4122190316	39.0016926430
H	9.4310282993	7.4172542459	38.9597983074
H	5.8933780525	5.9339656273	40.9143980627
H	7.8875273501	7.4204321749	40.9197904446
H	14.7149024928	3.7450418196	22.5766588010
H	15.4772922045	6.8044482692	25.5143707334
H	16.0920315335	4.9942992142	20.9254342041
H	16.8609100366	8.0459402580	23.8722763049
H	17.1505094094	7.1580603536	21.5575000176
H	10.6038878572	10.2963367277	25.4569086045
H	9.7654490797	7.2043508032	22.5761733049

H	9.9810645867	12.0315695840	23.8052433298
H	9.1932708194	8.9545590795	20.8958621573
H	9.2902453753	11.3570358081	21.4935750740
H	11.3727750536	7.0212048896	19.3023663283
H	13.1867136692	6.9404949663	23.2133628404
H	11.9824873768	9.4107414712	19.0779196300
H	13.7774805337	9.3245325123	23.0007278487
H	13.1539225922	10.5949134021	20.9385612973
H	15.6474771855	-3.2884028819	25.9027900586
H	12.1986765493	-4.1961940959	23.5028014957
H	17.1216731042	-4.2412602018	24.1492425923
H	13.6727695457	-5.1003130905	21.7133867890
H	16.1498756257	-5.1411915820	22.0433065452
H	6.7034543619	-2.6515265588	22.7239836419
H	10.1064715633	-5.2375284059	23.2214493235
H	6.5543059323	-3.2986364477	20.3389175171
H	9.9436432884	-5.9094979828	20.8398288817
H	8.1877329359	-4.9066634203	19.3638105784
H	14.6797393296	0.1658506727	24.7883636348
H	12.6807192055	-0.3569845660	21.0094925333
H	16.7180263139	-0.8697620344	23.8579909033
H	14.7287690602	-1.3914346387	20.0692530667
H	16.7643682155	-1.6503398267	21.4904144505
H	0.0846776764	2.2416822756	35.3757044169
H	3.3724769626	1.5898571088	38.0673546011
H	-1.0586583734	0.2230393529	36.2574459636
H	2.2143359439	-0.4238045974	38.9804548017
H	0.0081006834	-1.1275032168	38.0590209709
H	1.3631817894	9.6994581405	31.9256047551
H	3.8016207095	13.1180791808	32.9022934239
H	-0.1917406164	10.2746709446	33.7690549722
H	2.2348757290	13.7001461280	34.7540114027
H	0.2414539999	12.2763767624	35.1977279643
H	3.1906006929	-1.0114484917	28.8018087872
H	1.6607592923	1.5252463698	25.6737089702
H	2.1277600339	-2.9777479430	27.7337051357
H	0.5880878606	-0.4491684747	24.6026430502
H	0.8308034042	-2.7063634632	25.6139194130
H	2.9339084101	-1.3637813382	22.5456902707
H	5.5580950281	-3.3235067262	25.3527264737
H	2.1481378579	-3.5722262120	21.7555396981
H	4.7781219987	-5.5250158218	24.5612743849
H	3.0490548831	-5.6775479755	22.7603209887
H	11.3509987692	-1.0700384888	32.3220257300
H	8.8721196054	-2.8761850695	29.3019377918
H	10.3398320707	-2.5530549995	34.0437661187
H	7.8477626173	-4.3548823055	31.0236123794
H	8.5892093901	-4.2035064717	33.3966692358
H	12.3912197733	1.4172235385	32.9923489363
H	13.6882045239	5.3600263296	31.8839145079
H	13.7187400005	1.5688908955	35.0942793538
H	14.9972621871	5.5368946722	33.9988940021
H	15.0121722779	3.6335297339	35.6109723093
H	10.0012311709	-1.5082926032	23.0126148326
H	9.0324540354	0.2048250487	19.1731282324
H	11.5366843843	-3.1013779476	21.8875960919
H	10.5406723866	-1.4106320680	18.0492014968
H	11.7825551740	-3.0999174690	19.4232800329
H	7.6328319954	-0.9887398690	32.7296118172
H	8.9045681332	1.7763110801	35.7771375632

H	6.4663819848	-2.3394970140	34.4500728423
H	7.7265140655	0.4446187052	37.4986639380
H	6.5092745548	-1.6505847817	36.8378562926
H	-0.6780314493	7.0666619392	30.6329653277
H	1.7175614506	9.7824910255	28.3021683206
H	-2.3082764213	8.8839371858	31.0783763703
H	0.0927842783	11.6045826894	28.7511820969
H	-1.9232783680	11.1689999436	30.1526493615
H	5.5196633040	4.7452472737	18.0600412914
H	9.5328214560	6.3277872807	18.0190653974
H	4.8482060951	6.3921426623	16.3233358041
H	8.8608534818	8.0024819547	16.3014324060
H	6.5283216356	7.9950178575	15.4130278134
H	2.8807040488	7.4018257918	24.7502996870
H	0.7350358097	4.2865637311	26.8021125092
H	0.8313996375	8.8044736690	24.7328005243
H	-1.3051913849	5.7050811811	26.8233811814
H	-1.2667325083	7.9702671679	25.7913708848
H	2.4514395450	-0.2759452617	31.3872969606
H	4.8950399123	0.3457495319	34.8806490815
H	0.7914109234	-1.5112821816	32.7657033676
H	3.2721343508	-0.9501933331	36.2427105977
H	1.2290683937	-1.8949558119	35.1955305895
H	5.6840247551	-1.7807185285	31.4239706159
H	4.9887315866	-3.6293889400	27.5983928964
H	4.3619879494	-3.5642277386	32.5234412066
H	3.7659564158	-5.4865834133	28.7035420697
H	3.4569771371	-5.4736544164	31.1879613883
H	8.6064421564	8.6193656944	36.6404975495
H	6.7002436522	10.7702106199	33.4335299067
H	9.3810533999	10.7702457740	37.6207403829
H	7.4556514786	12.9163829712	34.4127161420
H	8.7928801426	12.9344102659	36.5244349432
H	2.6820100480	2.4096836469	23.5894890348
H	4.9432615081	1.4518434503	20.0544598753
H	0.8041592420	0.9979258211	22.7702781648
H	3.0514874384	0.0672615160	19.2051160017
H	0.9681108740	-0.1569792564	20.5682852976
H	7.9538891079	7.0734794496	20.9106475362
H	4.1695920508	7.2396338771	22.9510107427
H	6.9559794030	8.3127420392	18.9973178686
H	3.1491111534	8.3821114512	21.0062085619
H	4.5820227293	8.9918225253	19.0402550191
H	7.0278843103	9.4183101427	24.7549120355
H	2.8122824428	9.8024699222	25.5658047714
H	6.7333801085	10.6205482296	22.6147214722
H	2.5124230343	11.0260398685	23.4124782338
H	4.4747732651	11.4371437746	21.9308584398
H	3.9276946036	9.4359720305	33.1271965475
H	3.3406217626	6.6241616078	36.3291119983
H	4.5421613740	11.1944931031	34.7726810254
H	4.0946949652	8.3309760728	37.9508683108
H	4.6699811178	10.6330188921	37.1976904246
H	15.0121380875	3.1692479420	28.5947722967
H	14.1258012326	-0.9767521701	29.3389981231
H	16.1460353541	3.3286897337	30.8098568211
H	15.2752768524	-0.8260299503	31.5428150945
H	16.2696392851	1.3346934918	32.2909523806
H	11.6811672360	11.1614384980	31.3890117738
H	12.3722589752	7.0674116654	32.5249434163

H	14.0822721062	11.4065520611	30.7968237697
H	14.7849202024	7.3212985779	31.9548231328
H	15.6391110763	9.4727100672	31.0523403672
H	9.5718735506	13.0932508757	32.8058310769
H	7.2479775410	15.1231055804	29.7780785410
H	10.3345130960	15.2910638590	33.6660256663
H	8.0016334281	17.3063158696	30.6452598939
H	9.5721211215	17.4086431686	32.5908649615
H	0.8436388284	3.2069996320	29.0520629321
H	-0.0549689370	4.6780138466	32.9969693331
H	-1.4444299068	3.8256528502	28.2951560450
H	-2.3502221313	5.2925407092	32.2446192098
H	-3.0540368277	4.8596552642	29.8944936120
H	11.2775700785	10.5396003830	29.0677655172
H	7.8298886722	11.6158829669	26.7225351066
H	12.1537190785	12.8584441896	28.8544672539
H	8.7091431334	13.9329558987	26.4948693738
H	10.8592588127	14.5678869534	27.5745291749
